# The Origin of Life and Cellular Systems: A Continuum from Prebiotic Chemistry to Biodiversity

**DOI:** 10.3390/life15111745

**Published:** 2025-11-13

**Authors:** Jaime Gómez-Márquez

**Affiliations:** Department of Biochemistry and Molecular Biology, Faculty of Biology-CIBUS, University of Santiago de Compostela, 15782 Santiago de Compostela, Spain; jaime.gomez.marquez@usc.es

**Keywords:** life origin, life definition, prebiotic soup, origin of biodiversity, evolutionary forces

## Abstract

The origin of life remains one of the most profound and enduring enigmas in the biological sciences. Despite substantial advances in prebiotic chemistry, fundamental uncertainties persist regarding the precise mechanisms that enabled the emergence of the first cellular entity and, subsequently, the foundational branches of the tree of life. After examining the core principles that define living systems, we propose that life emerged as a novel property of a prebiotically assembled system—formed through the integration of distinct molecular worlds, defined as sets of structurally and functionally related molecular entities that interact via catalytic, autocatalytic, and/or self-assembly processes. This emergence established a permanent system–process duality, wherein the system’s organization and its dynamic processes became inseparable. Upon acquiring the capacity to replicate and mutate its genetic program, this primordial organism initiated the evolutionary process, ultimately driving the diversification of life under the influence of evolutionary forces and leading to the formation of ecosystems. The challenge of uncovering the origin of life and the emergence of biodiversity is not solely scientific, it requires the integration of empirical evidence, theoretical insight, and critical reflection. This work does not claim certainty but proposes a perspective on how life and biodiversity may have arisen on Earth. Ultimately, time and scientific inquiry will determine the validity of this view.

## 1. The Origin of Life: A Scientific Inquiry

“*I have no special talent. I am only passionately curious.*”

Albert Einstein

“*It is characteristic of science that the full explanations are often seized in their essence by the percipient scientist long in advance of any possible proof.*”

John Desmond Bernal

“*Reserve your right to think, for even to think wrongly is better than not to think at all.*”

Hypathia (c. 350-415 CE)

The origin of life (hereafter OoL) remains one of the most profound and unresolved scientific questions of our time [1]. Its investigation is inherently multidisciplinary, requiring contributions from biology, chemistry, physics, astronomy, geology, information theory, and philosophy. Although numerous hypotheses have been proposed to explain OoL, none has yet provided a fully satisfactory account [2].

As early as the 18th century, philosopher I. Kant suggested that life could only be understood under the assumption that it arises according to a design—not one attributed to an intelligent designer, but rather to nature itself. Building on this idea, many scientists have adopted a deterministic view of OoL, positing that life inevitably emerges when suitable conditions are met, whether on Earth or elsewhere in the universe. In support of this perspective, neurophysiologist G. Wald asserted: “Life in fact is probably a universal phenomenon wherever in the universe conditions permit and sufficient time has elapsed” [3]. Similarly, biochemist C. de Duve characterized the emergence of life as a “cosmic imperative” [4].

Several perspectives have been proposed to explain OoL on Earth. The theistic view, which invokes divine intervention, is rejected in this study in favour of a naturalistic explanation. Scientific inquiry must operate independently of theological frameworks. The goal is not to deny belief, but to ensure that scientific models remain within the bounds of methodological naturalism, which has proven to be a powerful tool for understanding the natural world. Another hypothesis, panspermia, suggests that life originated elsewhere in the cosmos and was transported to Earth [5]. While the panspermia hypothesis offers an intriguing possibility—that life, or its precursors, may have originated elsewhere in the cosmos and been transported to Earth—it ultimately does not resolve the fundamental question of how life began. Even if life were seeded from space, we would still be left without an explanation for the origin of that life, nor would we know where or under what conditions the initial biogenesis occurred. In this sense, panspermia shifts the problem rather than solves it. Therefore, while it remains a topic of legitimate scientific interest, it does not obviate the need to investigate natural, Earth-based scenarios for the OoL. The explanation considered most scientifically grounded here is that life emerged through the physicochemical transformation of inanimate matter into living matter, culminating in the formation of the first living system—the primordial cell. This event marked the beginning of biological evolution and the subsequent development of biodiversity, governed by natural laws and principles intrinsic to living systems [6].

Any scientific explanation for how life emerged from non-living matter must be grounded in known physical and chemical principles—such as thermodynamics, kinetics, molecular interactions, and conservation laws. These laws govern how atoms and molecules behave, interact, and organize under various conditions. They are universal and predictable, meaning they constrain how matter behaves under given conditions. Moreover, physical laws are deterministic and repetitive—they describe what must happen under certain conditions. Life, however, is dynamic and goal-directed—it involves regulation, adaptation, and purposeful behaviour (e.g., maintaining homeostasis, responding to stimuli, evolving). Living systems “steer” themselves toward survival and reproduction, which involves contingency, feedback, and control mechanisms—features not easily captured by static physical laws. Life involves emergent properties—like self-organization, information processing, and adaptive behaviour—that go beyond simple physical constraints. Physics and chemistry are necessary but not sufficient to explain life. We also need: biological principles (e.g., natural selection, genetic inheritance) and new elements from information theory (e.g., genetic coding, molecular signalling).

Research into OoL generally follows two complementary strategies. The bottom-up approach investigates the geochemical synthesis of biomolecules in primitive environments, while the top-down approach seeks to infer properties of ancient life from the conserved features of extant organisms [7]. It is widely accepted that the transition from non-living to living matter was a complex, multistage process. This progression likely involved the synthesis of biomolecules, the emergence of the genetic code, the development of metabolic pathways and energy conservation mechanisms, and ultimately the formation of protocells capable of self-replication and evolution [8,9].

To construct a logical and scientifically plausible explanation for this transition, one must rely on the laws of physics and chemistry, as living systems operate within these universal frameworks. In his seminal work What is Life? E. Schrödinger speculated that new physical laws might be required to explain life [10]. However, such laws have not been identified, suggesting that the principles of biology—rather than undiscovered physics—may be key to understanding life’s emergence and evolution.

While prebiotic chemistry provides the experimental foundation for understanding the transition from inert to living matter, the formation of the first living system marks the beginning of life’s history: the onset of evolution. Over geological timescales, biodiversity has undergone extensive transformation. Numerous species have gone extinct, while others, better adapted to changing biotic and abiotic conditions, have emerged. Nevertheless, the core molecular processes of cells—such as genetic information storage and expression, energy transduction, or central metabolic pathways—have likely remained conserved since the origin of the first living systems.

**Figure 1:**
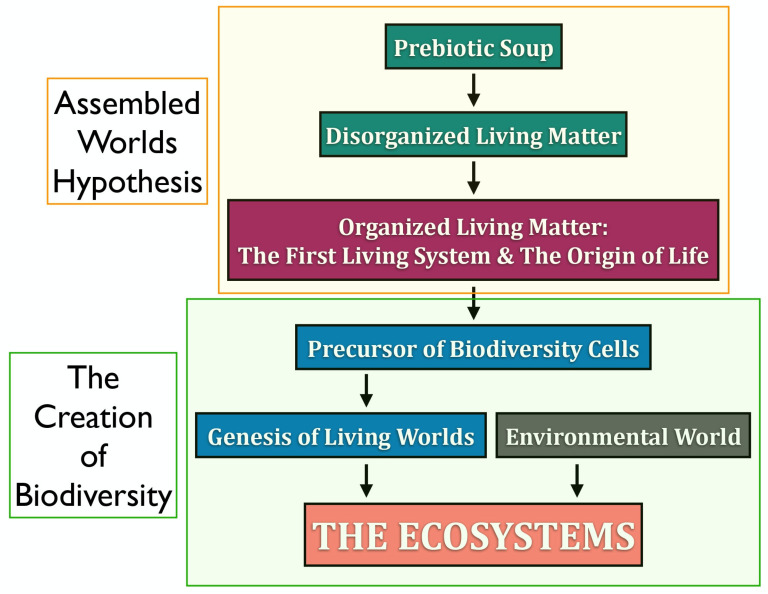
From the prebiotic soup to the creation of biodiversity. The transition from a prebiotic soup to the emergence of the first living system—initiating the life process—is partially explained by the Assembled Worlds Hypothesis. According to this framework, once the first living system was formed, it gave rise to precursor cells that seeded biological diversity. These early cells (the PoB cells), upon interacting with their surrounding environment, generated the three living worlds (AW, PW, EW) that evolved into the first ecosystems. This perspective emphasizes the dynamic interplay between nascent life forms and their ecological context, suggesting that life did not arise in isolation but as part of an integrated system of biochemical and environmental interactions.

In this work, we reflect on the concept of life and introduce a novel framework—the Assembled Worlds Hypothesis (AWH)—to explain the emergence of the first living system and the OoL. We also propose new ideas regarding the creation of biodiversity and the evolutionary forces that shaped its development. Figure 1 provides a broad overview of the evolutionary trajectory from the prebiotic environment to the emergence of complex ecosystems.

## 2. The Miracle of Life

“How can mindless molecules, capable only of pushing and pulling their immediate neighbours, cooperate to form and sustain something as ingenious as a living organism?” [11]. These words by P. Davies aptly capture the extraordinary and seemingly miraculous nature of the life process. Here, the term miraculous is not used in a religious sense, but rather to emphasize the improbability and wonder of biology’s foundational event. If one were to categorize the stages in the history of life, the moment of its emergence could be suitably termed the miracle stage, as the OoL represents “an extremely outstanding or unusual event, thing, or accomplishment”—one of the definitions of miracle in the Merriam-Webster Dictionary.

In the past century, F. Crick, co-discoverer of the DNA double helix and a founding figure in molecular biology, wrote: “... the origin of life appears at the moment to be almost a miracle, so many are the conditions which would have had to have been satisfied to get it going…” [12]. The astronomer F. Hoyle illustrated the same sense of improbability with a famous analogy: “A junkyard contains all the bits and pieces of a Boeing 747, dismembered and in disarray. A whirlwind happens to blow through the yard. What is the chance that after its passage a fully assembled 747, ready to fly, will be found standing there? So small as to be negligible, even if a tornado were to blow through enough junkyards to fill the whole Universe” [13].

Numerous hypotheses have been proposed to explain the OoL, yet none offers a fully satisfactory or complete account. From a genetic perspective, the RNA world hypothesis posits that prior to the emergence of life, there existed a prebiotic world in which RNA functioned both as an informational and catalytic molecule, capable of self-replication [14,15]. Although this hypothesis is supported by various scientific findings, it has also faced criticism from some researchers [16]. Alternatively, other scholars propose a prebiotic world characterized by a self-sustaining protometabolism, which serves as a heuristic alternative to the RNA world hypothesis [17]. From a thermodynamic standpoint, a provocative idea suggests that life exists because the law of increasing entropy drives matter to acquire life-like physical properties [18,19]. Furthermore, some authors have developed evolutionary theories of the OoL grounded in kinetics and thermodynamics [20]. Another notable proposal posits that mineral-associated organic microstructures may have evolved adaptively into cooperative ‘protolife’ communities [21].

Despite these diverse approaches, none has yet provided a comprehensive explanation for the OoL and the subsequent evolution of species. As J. Szostak pointed out: “To understand the origin of cellular life we have to understand the transition from a collection of biological building blocks to the assembly of a protocell capable of growth, division and Darwinian evolution” [22]. We will probably never know exactly what the OoL was like, but thanks to scientific research and theoretical considerations we can get much closer to the truth of what happened billions of years ago.

In the history of life, OoL represents the foundational event—both the most momentous and the most enigmatic. Two additional biological miracles occurred at the dawn of life on Earth, DNA replication followed by cell division and the beginning of evolution, shaping the trajectory of biodiversity for millions of years thereafter. The first division of the primitive cell, likely resulting in two daughter cells, marked the inception of biological reproduction and the continuity of vital processes. It seems likely that cell division in primitive cells was affected by imperfect duplication of genetic material and insufficient segregation fidelity. Without cell division, life could not be sustained across space and time; living systems would eventually vanish, and the postbiotic soup would need to generate a new cell once again. The capacity for reproduction ensured the continuity of species and their evolution into new, better-adapted biological systems.

The third miracle was the evolution of species. The genetic program embedded within those early cells was not static—it possessed the intrinsic capacity for mutation. This potential for variation, coupled with replication, gave rise to the evolutionary diversification of species. Later in this paper, we will briefly examine the evolutionary mechanisms that contributed to the emergence of biodiversity.

## 3. Information, System-Process Duality, Emergence and Negentropy

In addition to the evolutionary dimension that permeates all biological processes, four fundamental components—information, emergence, system–process duality, and negentropy—are intrinsically linked to the phenomenon of life. These elements should be considered not only in efforts to define life, but also in determining its existence. Accordingly, any organic entity that demonstrably exhibits all four characteristics may be reasonably regarded as a living system.

### 3.1. Information and Life

Biological information refers to the data encoded, transmitted, and interpreted within living organisms to sustain and regulate life processes. The encoding and processing of inheritable information are mediated by genetic systems (e.g., DNA and RNA), which store executable instructions for the construction, maintenance, and reproduction of organisms. In all known life forms, this information is embedded in a genomic program that is subject to mutation—alterations that may unpredictably modify the original informational content and yield a revised program. When expressed, such modifications can result in phenotypically distinct organisms. The evolutionary process on Earth is fundamentally driven by these changes in genetic programs, which may generate novel biological information or, conversely, produce dysfunctional outputs with maladaptive or deleterious consequences.

Traditional definitions of information, such as those derived from Shannon entropy, conceptualize it as statistical uncertainty [23]. However, in biological systems, information is inherently functional. It evolves over time, prescribes specific actions (e.g., gene expression), is differentially expressed across spatial contexts (e.g., cellular environments), and temporal stages (e.g., embryogenesis), and is encoded symbolically (e.g., codons representing amino acids). Thus, biological information transcends thermodynamic possibility—it is instructional and executable, akin to software operating within a computational system [24].

Living systems actively acquire, process, and utilize environmental information to adapt to changing conditions, maintain homeostasis, and pursue intrinsic functional objectives. In this context, the emergence of information-driven mechanisms represents a pivotal milestone in the process of abiogenesis [25]. M. Barbieri further emphasizes that biological information is not merely statistical, but semantic—it carries meaning and function. He introduces the concept of organic codes, symbolic systems such as the genetic code, splicing code, and histone code, which govern biological processes and suggest that life is regulated not only by physical laws but also by symbolic conventions [26].

Can physicodynamics encode information? Physicodynamics encompasses the laws and dynamics governing physical systems—such as thermodynamics, kinetics, and quantum mechanics. While these frameworks describe molecular interactions and energy transformations, they do not inherently encode functional information. That is, they delineate what can happen, but not what should happen in a goal-directed or context-sensitive manner. Notably, recent research suggests that information dynamics in early life may have emerged from environmental cycles (e.g., day–night rhythms), which imposed selective pressures and contributed to the organization and evolution of functional information [27].

When a mixture containing DNA, DNA polymerase, primers, nucleotides, and suitable buffer components is subjected to thermal cycling conditions in vitro—as in the polymerase chain reaction (PCR)—the system autonomously amplifies a specific DNA fragment defined by the primer sequences. This process unfolds without the need for an external algorithm or conscious intervention. The molecular constituents interact according to their intrinsic chemical affinities and thermodynamic properties, resulting in the emergence of a highly ordered and functional outcome: the exponential replication of a defined DNA sequence. This phenomenon exemplifies self-organization, wherein complex behaviour arises from local interactions governed by physical laws, absent centralized control or symbolic instruction. However, PCR is not an algorithmically driven process; it lacks the symbolic interpretive machinery characteristic of living systems. It does not rely on “prescriptive information”—that is, sequences or codes that explicitly dictate a series of operations to achieve a specific functional goal [28].

In the context of the OoL, this raises a fundamental question: how did systems transition from purely physicochemical self-organization to algorithmically controlled, information-driven biochemistry? This remains one of the central unresolved questions in OoL research. While the answer is currently unknown, it is conceivable that multiple layers of self-organizing phenomena overlapped, eventually generating an internal environment sufficiently complex to give rise to the first living system. In this view, life emerged as a consequence of a multi-tiered process of self-organization.

For life to arise spontaneously as an emergent property of matter, the mere formation of complex organic molecules is insufficient; these molecules must also be organized in a manner that encodes functional instructions. This is exemplified by the central dogma of molecular biology, wherein nucleotide sequences in DNA or RNA prescribe the assembly of amino acids into proteins with specific biological functions. Such a system implies the existence of a symbolic, rule-based framework capable of interpreting and executing these instructions—namely, the integration of genetic information with transcription and translation mechanisms.

Although this molecular machinery appears exceedingly complex, it can operate autonomously in a simple in vitro setting, devoid of cellular organization. Even intricate processes such as recombination can occur during PCR via G-quadruplex formation [29]. The key may lie in uncovering the origin of the genetic code and its interpretation through translation [30,31]. In other words, understanding how information encoded in symbolic units (triplets or codons) was transformed into functional proteins.

Whatever the ultimate answer to the enigma of how codes, symbols, or algorithms governing biological processes were established, one certainty remains: the emergence and perpetuation of genetic information flow was the foundational event upon which the first living systems—and thus life itself—were built.

### 3.2. System-Process Duality

The system–process duality refers to the intrinsic relationship between life as a dynamic process and the living system as its structural substrate [32]. Life cannot exist independently of the system in which it manifests, nor can the system be considered living in the absence of the life process. Together, they constitute an inseparable and mutually constitutive unity. This duality is central to both scientific and philosophical understandings of life, implying that life is not merely a matter of molecular composition, but rather of the temporal and spatial interactions among those molecules that sustain a dynamic, far-from-equilibrium state. Importantly, this duality does not represent a dichotomy or opposition, but a unified phenomenon composed of two interdependent aspects. Life and the living system are thus two facets of a single entity—only in conjunction do they attain functional coherence and biological significance.

Much like the classical ‘chicken-or-egg’ dilemma, one may ask: which preceded the other—the system (i.e., the first cell) or the process (i.e., life itself)? To address this question, it is essential to examine the OoL. At that primordial stage, when the system was only beginning to take shape, the life process had not yet emerged. Nevertheless, with the building of the first living system, the life process manifested as an immediate and intrinsic property of that system. From that moment onward, an inseparable relationship was established between the system—the living organism—and the process—life itself. This marked the emergence of the system–process duality, effectively rendering the initial dilemma obsolete. Once this original system acquired the capacity for division and, consequently, for evolution, the generation of biological diversity was set into motion.

### 3.3. Life as an Emergent Property

An emergent property refers to a novel and unpredictable characteristic that arises from the ordered interactions among components within a system. Emergence can be conceptualized as the outcome of cooperative dynamics among autonomous elements, which collectively give rise to new macroscopic structures and systemic behaviours [33].

Life is widely recognized as an emergent property of complex molecular systems. It arises from the intricate interplay among non-living molecular constituents—such as proteins, nucleic acids, lipid membranes, metabolic networks, and molecular receptors—which, in isolation, lack the capacity to sustain life. However, when these components are assembled into a highly ordered and functionally integrated structure, such as a cell, they collectively exhibit properties that are irreducible to their individual parts. These emergent properties include reproduction, evolution, homeostatic regulation, and adaptive responsiveness [34,35]. An illustrative analogy can be drawn from the digestive process, which may also be considered an emergent property of the digestive system. No single anatomical component—whether the mouth, oesophagus, stomach, or intestines—is capable of performing digestion independently. Rather, digestion arises from the anatomical organization and functional coordination of these components, mediated by molecular signals such as hormones. Only when this integrated system is operational does the process of digestion emerge.

It is hypothesized that life emerges as a systemic property once all requisite components of living matter are spatially and temporally organized in a coherent and sequential manner, thereby establishing the inseparable duality of system and process. This concept is foundational in systems biology and complexity science, helping to explain why the mere assembly of biological molecules does not automatically result in a living system—it is the organization and interaction that matter. Despite significant advances in molecular biology and biochemistry, the precise mechanisms by which life emerges from the interactions of cellular components remain elusive. The inability to synthetically recreate life in vitro underscores our limited understanding of how disordered molecular assemblies transition into organized, living systems.

Self-organization plays a pivotal role in the emergence of life, particularly in the formation of prebiotic structures preceding the OoL [36,37]. The spontaneous emergence of order among molecular ensembles—without external direction and beyond the explanatory power of their isolated components—has been proposed as a defining feature of life’s origin [38]. It is reasonable to posit that life could not have arisen until inanimate matter, initially disordered within the so-called “primordial or prebiotic soup,” achieved a stable self-organized configuration [39].

Furthermore, some theoretical frameworks suggest that the emergence of life represents a transition from second-order emergence—characteristic of diachronic self-organization in energy-dissipative systems—to third-order emergence, which involves transgenerational biasing mechanisms akin to biological development and evolutionary processes [40].

### 3.4. Entropy, Negentropy and Life

Living systems are distinguished by their ability to resist entropy and sustain highly ordered states over time [41]. E. Schrödinger introduced the concept of negative entropy (or “negentropy”) proposing that living systems maintain internal order—characterized by low entropy—by exporting disorder to their surroundings [10]. He famously described life as a system that “feeds on negative entropy,” highlighting the paradoxical nature of biological order in a thermodynamically disordered world.

In the context of life, negentropy is a defining feature. Living organisms continuously import energy and matter from their environment (e.g., nutrients, sunlight) and use these inputs to build and maintain complex structures, regulate internal conditions, and perform coordinated functions. The strategies evolved by organisms to acquire and utilize energy have been among the primary drivers of biological evolution [42]. This active maintenance of order is what allows life to persist in a universe governed by the second law of thermodynamics.

The inherent tendency of living matter to degrade into inert, disordered states is counteracted by the continuous generation and maintenance of negative entropy. This capacity to resist entropy and maintain organized complexity is a defining feature of life, underscoring the dynamic equilibrium necessary for biological persistence. Through processes like metabolism, repair, and reproduction, organisms counteract the natural tendency toward equilibrium and decay. Importantly, this negentropic behaviour does not violate thermodynamic laws; rather, it is enabled by open-system dynamics, where living systems exchange energy and matter with their surroundings.

Negentropy is also closely linked to information and emergence. The genetic program of an organism encodes instructions that guide the construction and maintenance of ordered structures. Emergent properties—such as consciousness, metabolism, or homeostasis—arise from the organized complexity sustained by negentropic processes. In abiogenesis, the emergence of negentropic behaviour may have marked a critical transition from disordered molecular ensembles to proto-biological systems capable of maintaining internal order. The appearance of self-organizing, energy-processing structures in the prebiotic environment likely played a pivotal role in the OoL [43].

Reproduction represents a fundamental victory of life over entropy, as it generates a new vital order from pre-existing biological systems [6]. Upon birth, an organism begins a temporal journey—a race against the arrow of time—ultimately culminating in death, in accordance with the second law of thermodynamics. In the existential struggle described by Boltzmann, there are two simultaneous victors. Life prevails through reproduction, which perpetuates biological order and complexity. Entropy, however, also advances, as the metabolic activity of living organisms and the decomposition of biological matter after death contribute to the overall increase in the entropy of the universe. This dual outcome underscores the paradox at the heart of biological existence: while life locally defies entropy through organization and replication, it inevitably contributes to the entropic progression of the cosmos.

## 4. The Importance of Defining Life

Scientific concepts serve not merely as linguistic labels but as foundational tools for inquiry. Their definitions must encapsulate essential attributes to ensure precision and avoid ambiguity. In gravitational physics, for example, the concept of gravity must be clearly defined to distinguish between Newtonian and relativistic frameworks. In robotics, the definition of a robot influences design, functionality, and ethical considerations. Similarly, in OoL research, the absence of a universally accepted definition of life presents both a philosophical challenge and a practical obstacle. A universally accepted definition of life would not only hold academic and theoretical value but would also be highly beneficial in fields such as astrobiology, synthetic biology, artificial life, and the study of viral acellular systems.

The key question is: how can we investigate the OoL without first establishing what life is? If we argue that a definition of life is unnecessary for studying its origin, then we must ask—what origin are we attempting to uncover? This straightforward line of reasoning underscores the importance of having a conceptual framework for life to meaningfully explore its beginnings. While the absence of a universally accepted definition does not preclude laboratory experimentation, it does highlight the philosophical and theoretical challenges inherent in OoL research.

Despite extensive interdisciplinary efforts, no single definition of life has achieved universal consensus [32,44,45,46,47,48,49]. Definitions vary depending on whether the focus is biochemical, thermodynamic, informational, or evolutionary. This lack of consensus reflects the complexity of life as a phenomenon and the diversity of perspectives across biology, philosophy, and artificial life studies. Yet, a coherent conceptual framework is indispensable. Without it, experimental efforts to recreate life from prebiotic conditions risk being directionless or inconsistent. A working definition—while perhaps provisional—can guide the design of experiments, the interpretation of results, and the integration of findings across disciplines. Among the many existing definitions of life, I have chosen to adopt two ([32,48]), both of which I previously proposed. The second and more recent definition [48] represents an evolution of the first and serves as the conceptual foundation for describing the characteristics of the earliest living system.

In the context of OoL research, efforts to reconstruct a living system from plausible prebiotic conditions should yield, as a theoretical and experimental target, an organic system grounded in carbon-based chemistry. As previously discussed, to determine whether a newly constructed organic system qualifies as “living” within the context of OoL studies, it must meet several foundational criteria. First, the system should be thermodynamically open, capable of exchanging energy and matter with its environment—this reflects the metabolic processes essential for energy acquisition and transformation. Second, it must exhibit structural order and be functionally organized, with its components contributing to the system’s persistence and coherence; this aspect underscores the importance of negentropy, or the maintenance of order against entropic decay. Third, the system should encode heritable information—a genetic program that enables reproduction and evolutionary adaptation over time. Fourth, the system should demonstrate adaptive responses to environmental fluctuations. These criteria collectively provide a framework for evaluating whether a synthetic or reconstructed system can be meaningfully considered “alive” in experimental OoL contexts.

Regardless of what may ultimately constitute the most accurate definition of life, I wish to emphasize—and maintain as fundamental—the importance of having a clear conceptual understanding of life in any research aimed at investigating its origin. While experimental approaches can proceed without a universally accepted definition, such clarity is essential for framing meaningful questions and interpreting results within a coherent theoretical context.

## 5. The Fundamental Enigma: The Origin of the First Living System

If life is accepted as an emergent property, then the central challenge in OoL research is to elucidate the mechanisms by which the first living system came into being. Although both the scientific literature and popular science frequently refer to the search for life on other planets or the artificial synthesis of life in laboratory settings, these endeavours are fundamentally directed toward identifying or constructing living systems—whether extraterrestrial or synthetic—that exhibit the essential characteristics of life. For example, astrobiologists search for biosignatures or fossilized traces of life beyond Earth based on our current knowledge of life on Earth, recognizing that life itself does not fossilize or leave traces, but only the systems that sustain it.

Consequently, the primary objective of OoL research is to understand how the first living system could have originated under the environmental conditions presumed to have prevailed on the early Earth. Should scientists succeed in constructing such a system in vitro, life would not need to be externally introduced or induced; rather, it would emerge spontaneously because of the system’s intrinsic organizational properties.

## 6. The Assembled Worlds Hypothesis

Understanding the transition from prebiotic chemistry to modern biochemistry is tantamount to elucidating how inanimate matter gave rise to the first living system. How, precisely, did this system emerge from the primordial soup? While definitive answers remain elusive, ongoing advances in prebiotic chemistry are progressively narrowing the gap between speculative hypotheses and plausible reconstructions. Research in OoL science oscillates between empirical data and theoretical models shaped by biological intuition.

AWH posits that the first living system arose within the primordial prebiotic environment through the ordered and organized fusion of distinct molecular domains: the metabolic world, the biomolecular world, and the supramolecular world. Within this framework, a molecular world is defined as a set of structurally and functionally related molecular entities that interact via catalytic, autocatalytic, and/or self-assembly processes. Once the first living system was assembled through the integration of these molecular worlds, life emerged as a novel property of that system, thereby establishing the enduring system–process duality. Upon acquiring the capacity to replicate and mutate its genetic program, this primitive cell initiated the evolutionary process, ultimately leading to the diversification of life and the emergence of ecosystems. Although the existence of such worlds in the prebiotic Earth remains speculative—as is much of what pertains to events nearly four billion years ago—indirect evidence may support their plausibility.

The key to the assembly of molecular worlds lies, first, in the genesis of life’s fundamental molecules within what is referred to here as the Mysterious Earthly Place (MEP), and second, in the process of molecular convergence. The abbreviation MEP designates the location(s) where life first emerged. This term is used metaphorically to represent the unknown site of the OoL and is justified by three considerations: (i) the precise location of the OoL remains a mystery; (ii) it acknowledges that life originated on Earth; and (iii) the exact geographical or geological context of this emergence is currently unknown. The use of this terminology is intended to evoke the enigmatic nature of this foundational event without implying any specific spatial constraints or environmental conditions. Furthermore, for the purposes of this study, the term “prebiotic soup” is employed without presupposing any particular environmental context—such as a terrestrial pond or marine setting—as the locus of life’s emergence.

**Figure 2:**
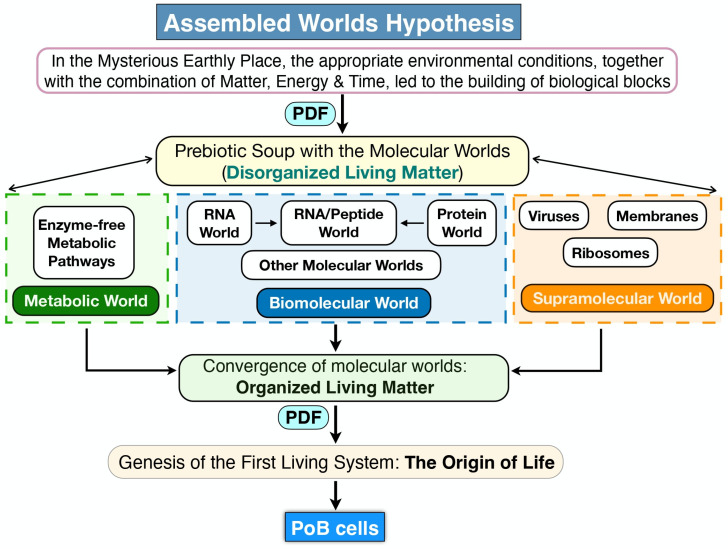
The Assembled Worlds Hypothesis. In the MEP the interplay of matter, energy, and time initiated the formation of a primordial chemical environment—commonly referred to as the “prebiotic soup”—which eventually gave rise to distinct molecular worlds: metabolic, biomolecular, and supramolecular. These worlds, initially characterized by low organization and minimal interconnectivity, harboured the molecular constituents essential for the emergence of the first protocell. This stage represents the assembly of fundamental biological building blocks. The transition from disorganized living matter—comprising diverse biomolecules that did not yet engage in coordinated interactions—to organized living matter, where biomolecules began to interact systematically, likely involved two critical processes: the synthesis of novel biomolecules and an increase in their local concentrations, which facilitated molecular interactions. At a certain point, this organized living matter became encapsulated within a membranous structure, giving rise to the first living system capable of sustaining its own metabolism. This primitive system could synthesize molecules and harness energy, feature an incipient genome, and employed RNA-mediated protein synthesis with catalytic functions. All molecular reactions and interactions were driven by physicochemical dynamic forces. The formation of this first cellular biological entity marks the OoL, as life arouse as an emergent property of this organized, self-sustaining system.

Within this conceptual framework, the AWH outlines a sequential pathway from raw matter and energy on early Earth to the emergence of the first living system (Figure 2). It comprises two distinct phases:

1. Priming Phase—characterized by the formation of disorganized living matter and its differentiation into several distinct prebiotic molecular worlds.
2. Assembly Phase—involving the convergence of these molecular worlds into a unified and functionally coherent living system.

Several contemporary studies exhibit conceptual parallels with the AWH. Notably, Assembly Theory, developed by Cronin and Walker [50], investigates the emergence of complex molecular architectures through historical assembly pathways. While this framework emphasizes emergence and structural organization, its primary focus lies in quantifying molecular complexity rather than integrating diverse molecular domains. The Eco-evolutionary Origins of Life hypothesis [51] proposes that life emerged via eco-evolutionary feedback mechanisms, emphasizing systems-level interactions and the adaptive complexity of prebiotic environments. This perspective aligns with the notion of life arising from coordinated molecular domains. Additional contributions have explored the transition from abiotic chemistry to biological systems, emphasizing thermodynamic constraints and bioenergetic principles [52]. These studies support the view that life is a systemic property arising from organized molecular interactions. Finally, the Autopoiesis and Synthetic Biology Framework proposed by Luisi [53] investigates the roles of self-organization and compartmentalization in the origin of cellular life. This framework strongly reinforces the concept of life as an emergent property of integrated molecular systems.

### 6.1. The Priming Phase: The Genesis of Molecular Worlds

Prebiotic chemistry aims to elucidate how the intricate biochemistry that characterizes contemporary life could have emerged from simpler chemical processes in the distant past [54,55,56]. The foundational milestone of this field is commonly attributed to the Miller–Urey experiment [57], which demonstrated that organic molecules can be synthesized under conditions presumed to resemble those of the early Earth. Since then, prebiotic chemistry has substantially advanced our understanding of how the organic matter that preceded the first living systems may have originated [58,59,60,61].

In addition to the traditional bottom-up and top-down approaches—which extrapolate current biological paradigms into the past—there is also a bottom-up strategy that explores alternative prebiotic chemistries [62]. These different prebiotic chemistries may have played a role in the OoL but are no longer preserved in extant biology [58]. Regardless of the specific chemical pathways involved, if we accept that life originated from inanimate matter somewhere on Earth, it follows that living matter must have formed prior to the emergence of the first living system.

In the MEP, under favourable conditions—combining sources of matter (e.g., soil, rocks, oceans, atmosphere) and energy (e.g., solar, geothermal)—and over vast timescales, the processes leading to the OoL began [63]. The primordial prebiotic soup likely consisted of an aqueous medium wherein organic and inorganic compounds interacted, giving rise to increasingly complex molecules. These abiotic processes were governed by fundamental physicochemical driving forces (hereafter PDFs). This term broadly encompasses the ensemble of natural forces and conditions that may have facilitated the formation of complex organic molecules in the prebiotic environment. These include energy sources such as geothermal heat, solar radiation, and redox gradients; chemical gradients that promoted molecular transport and concentration; temperature fluctuations that influenced reaction kinetics and phase transitions; intrinsic chemical reactivity among functional groups of organic molecules; and self-organization phenomena, including molecular crowding, phase separation, and the spontaneous formation of supramolecular structures. Acting synergistically under non-equilibrium conditions, these factors likely played a central role in the emergence of the first living systems. Rather than positing a single deterministic pathway, this perspective supports a systems-level framework in which life emerged from the dynamic interplay of diverse physicochemical processes.

Three major stages preceded the emergence of the first living system. First, within the initial broth, organic matter was synthesized from small inorganic precursors. This organic matter underwent spontaneous transformations, yielding simple biomolecules. Second, as the chemical complexity and concentration of the soup increased, the first biological building blocks—defined here as structurally or functionally analogous to those found in modern organisms—began to form. These included monosaccharides, amino acids, fatty acids, phospholipids, and nucleotides. Thirdly, these molecules gradually evolved into more complex structures and processes, culminating in what we refer to here as “molecular worlds.” The subsequent formation of phospholipid vesicles, the emergence of enzyme-free reaction pathways, or the synthesis of macromolecular assemblies (e.g., protoribosomes, primitive membranes) marked the transition to disorganized living matter, in which new molecular interactions and processes began to emerge, although still unconnected or only partially connected. At this stage of disorganized living matter, the fundamental molecular components of cellular life were already present. However, a critical feature of living systems remained absent: order, defined as the internal organization and coordination of all molecular components. The establishment of such order required the formation of a membrane capable of encapsulating these molecular worlds, thereby enabling their interaction—initially at the molecular level and subsequently at the cellular level. Through spontaneous self-organization of the entire system, life ultimately emerged.

The synthesis of the first biomolecules and complex macromolecules gave rise to several initially independent molecular worlds: (i) The Biomolecular World: involving informational and catalytic macromolecules as well as other molecular components of the living matter; molecules such as catalytic RNAs or ribozymes, lipid bilayers formed from phospholipids, peptides, and nucleic acid–protein complexes—potential precursors of primitive viruses—began to emerge; (ii) The Metabolic World: consisting of reaction networks capable of energy transformation and molecular synthesis (the first enzyme-free metabolic pathways began to emerge); and (iii) The Supramolecular World: comprising self-assembled structures such as membranous vesicles, ribosomes, viruses and other macromolecular structures. These worlds constituted the first forms of living matter, albeit in a disorganized state and therefore not yet alive. Once the initial organic molecules were synthesized in the primordial broth, the emergence of macromolecules and supramolecular structures marked the beginning of the evolutionary trajectory of the prebiotic molecular worlds.

The origin of these molecular worlds within the MEP can be attributed to increasing molecular complexity and the emergence of novel interactions. These interactions led to the formation of macromolecular structures, the appearance of processes such as replication and autocatalysis, and the concatenation of chemical reactions into primitive metabolic pathways. All these developments were governed by PDFs and, in many cases, were driven by chemical determinism, which played a fundamental role in the evolution of the prebiotic broth.

Before moving on to describe molecular worlds, I would like to give an example of the integration of molecular worlds in a “laboratory-created soup.” I am referring to IVTT (in vitro transcription and translation) kits [64]. These kits are a cell-free system that generates RNA from a DNA template and then uses that RNA to synthesize proteins in a single tube. We could say that they are equivalent to what I named as disorganized living matter. Isn’t it surprising how such complex processes as transcription and translation can nevertheless take place in a test tube in a complex mixture of biomolecules, salts, and water? This kit is an example of how different molecular elements, which would form part of molecular worlds, self-organize to manufacture proteins in vitro. Could it be that if we added glucose at the same time, it would be converted into pyruvate, generating ATP for transcription and translation? This could be another example, this time hypothetical but possible, of the convergence of the three molecular worlds.

#### 6.1.1. The Biomolecular World

Within the biomolecular world, it is conceivable that several molecular worlds coexisted–namely, the RNA world, the protein world, and a third world encompassing other molecular entities such as lipids and carbohydrates; this latter world could potentially be further subdivided into more specific molecular categories. It is plausible that, during their nascent stages, these molecular worlds operated largely independently, exhibiting minimal inter-world interactions. This conceptual framework underscores the likelihood that, in the prebiotic environment, molecular interactions initially occurred preferentially among chemically similar species.

Experimental evidence supports the plausibility of such biomolecular world in the MEP. In this context, a notable study has demonstrated a shared origin for RNA, protein, and lipid precursors within a cyanosulfidic protometabolic framework [17], suggesting that the fundamental biomolecules required for the emergence of life could have originated concurrently within the same prebiotic environment. Undoubtedly, the prebiotic soup contained not only these molecular worlds but also a wide array of other organic molecules and inorganic compounds that contributed to the emergence of these primordial systems. While the conceptualization of such a biomolecular world remains speculative, it is nonetheless evident that the structural precursors of the biomolecules that would eventually constitute the first living cells must have formed during this period.

A very interesting and important aspect for understanding the OoL is how molecular chirality emerged [65]. Many biomolecules—particularly amino acids, which are almost exclusively left-handed (L-enantiomers), and sugars in nucleic acids, which are right-handed (D-enantiomers)—exhibit chirality. This phenomenon of homochirality remains one of the central mysteries of life. In this hypothetical biomolecular world undergoing constant transformation, the chirality of biomolecules was established through a mechanism that remains unidentified. This process—potentially encompassed within PDFs—must have preceded the emergence of the first nucleic acids and proteins, as chirality critically influences protein folding and the structural configuration of nucleic acids.

##### The RNA World

The concept of the RNA world was first proposed by W. Gilbert in 1986 and has since been extensively developed by numerous researchers [66,67]. This hypothesis is grounded in four fundamental properties exhibited by certain RNA molecules: (i) the ability to encode genetic information within their sequences, (ii) the capacity for self-replication, (iii) intrinsic catalytic activity, and (iv) the potential to evolve under selective pressures [68]. The RNA world hypothesis provides a plausible starting point for life’s emergence, bridging the gap between simple chemistry and complex biology. It supports the idea that life could begin with a self-replicating, evolving system, laying the foundation for Darwinian evolution long before cells existed.

Nevertheless, while the emergence of self-replicating, information-bearing RNA molecules is widely regarded as a pivotal milestone in the OoL, it is unlikely that RNA alone could have driven the transition from chemistry to biology. The OoL required more than information storage and catalysis; it also depended on the availability of energy sources, the synthesis of diverse biomolecules, and the formation of compartmentalized structures. Thus, the RNA world must be understood as part of a broader network of interacting molecular domains—including metabolic, supramolecular, and energetic systems—that collectively enabled the emergence of the first living entities. This integrated perspective supports a systems-level view of the OoL, in which life arose from the dynamic interplay of multiple molecular worlds within the prebiotic soup.

The RNA world hypothesis proposes that RNA-based life emerged from chemically synthesized nucleotides present in the prebiotic soup. These nucleotides eventually polymerized to form the earliest RNA molecules, giving rise to a self-sustaining RNA-based system capable of both information storage and catalytic activity. However, the precise mechanisms by which nucleosides in the prebiotic environment underwent phosphorylation to become ribonucleotides, and subsequently polymerized to yield functional RNA, remain unresolved. These gaps highlight current limitations in our understanding of prebiotic chemistry and underscore the challenges in reconstructing plausible pathways for the OoL [68,69].

As previously discussed, RNA possesses a remarkable range of functional capabilities: it can store genetic information, as demonstrated by the existence of RNA viruses; it exhibits catalytic activity, exemplified by ribozymes and its role in peptide bond formation; it plays a central role in translation through mRNA, tRNA, and rRNA; it regulates gene expression via small non-coding RNAs such as microRNAs; and it serves as a biochemical precursor in the synthesis of DNA. Moreover, in nucleotide metabolism, ribonucleotides are synthesized prior to deoxyribonucleotides, and several essential coenzymes—including NAD^+^, NADP^+^, and FAD—contain ribonucleotide moieties. Although DNA functions as the primary genetic material in most extant organisms (with the exception of certain RNA viruses), RNA is widely believed to have preceded DNA in early evolutionary history due to its structural and functional versatility.

Taken together, these properties underscore the central role of RNA in both modern biological systems and early biochemical evolution, reinforcing its proposed primacy in the OoL [70]. Undoubtedly, the most compelling evidence supporting the RNA world hypothesis is RNA’s dual functionality—as both an informational molecule and a biocatalyst capable of catalysing the replication of RNA genomes [71,72].

##### The Peptide World

The interactions between proteins and nucleic acids are fundamental to the molecular processes that define life. Replication, transcription, translation, and gene regulation constitute the core mechanisms through which biological information is expressed, and evolutionary change is enabled. Accordingly, the presence of proteins in the prebiotic environment—and their subsequent interactions with RNA molecules—represented a pivotal advancement in the OoL. The ensemble of amino acids and peptides/proteins synthesized under prebiotic conditions is referred to as the peptide world.

Multiple mechanisms have been proposed to account for the formation of peptide bonds between amino acid monomers under plausible prebiotic conditions [73]. For example, the discovery of boron-containing minerals in ancient sedimentary deposits supports the hypothesis that boron may have catalysed polypeptide synthesis in early Earth environments [74]. Notably, such conditions may have also facilitated RNA formation and its interactions with emerging peptides. Additionally, recent studies suggest that the catalytic properties of phosphate chains, combined with the crystalline organization observed in amino acid–phosphoric acid salts, could have enabled the synthesis of short oligopeptides within mildly acidic hydrothermal settings [75]. Given that phosphates are essential components of nucleotides—the building blocks of nucleic acids—it is reasonable to infer that they played a critical role in the co-emergence of both the RNA and protein worlds.

Further research has proposed that amino acids, in combination with α-hydroxy acids (e.g., citric acid, a Krebs cycle intermediate, and lactic acid, a fermentation product), may have polymerized into polypeptides through cycles of hydration and dehydration under fluctuating temperature conditions, independent of RNA or ribosomal machinery [76,77]. Regarding the transition from short peptides to functional proteins, recent findings suggest that proteins may have originated through a combinatorial process involving the modular assembly of short peptide segments or “building blocks” [78].

##### The RNA-Peptide World

The coexistence of the RNA world and the peptide world likely led to the emergence of a hybrid RNA–peptide world, representing a critical transitional phase in the OoL. Initially, these two molecular domains may have functioned independently, driven by autocatalytic reactions or mineral-mediated catalysis within the prebiotic environment. Over time, however, their integration likely increased in complexity, culminating in the formation of primitive molecular systems in which RNA molecules catalysed peptide synthesis. One of the enduring questions in OoL research is how this RNA-centric system transitioned into one dominated by proteins as the primary catalysts, with RNA assuming predominantly informational and regulatory roles.

Recent studies have provided compelling evidence that non-canonical RNA bases—still present in modern transfer and ribosomal RNAs and considered molecular fossils of the RNA world—can facilitate peptide synthesis directly on RNA scaffolds. These findings support the hypothesis that an RNA–peptide world preceded the emergence of ribosomal translation and may have served as a precursor to the modern protein synthesis machinery [79,80,81].

The defining feature of this RNA–peptide world was likely the establishment of translation—the synthesis of proteins from genetic templates. It is plausible that RNA molecules initially catalysed peptide bond formation in the prebiotic soup, although alternative chemical systems may also have contributed. This hypothesis is consistent with current biological knowledge, as ribosomal RNA is responsible for catalysing peptide bond formation in contemporary cells. Once the prebiotic environment became enriched with both RNA and peptides, the RNA–peptide world would have emerged as a natural consequence [82,83,84,85].

##### Other Molecular Worlds

The significance of RNA and peptides in the prebiotic soup lies in their foundational role in the emergence of the central dogma of molecular biology—namely, the origin of the genetic code, gene expression, and protein synthesis. However, they were not alone in the prebiotic soup. In fact, the molecular complexity of the prebiotic soup increased further with the emergence of metabolites associated with the onset of primitive metabolic processes.

In addition to amino acids, peptides, nucleotides, and nucleic acids, the prebiotic environment harboured a wide variety of organic and inorganic molecules that contributed to the OoL. Among these, lipids deserve particular attention due to their critical role in the formation of the earliest biological membranes and membranous vesicles. These amphiphilic molecules were capable of self-assembly under plausible prebiotic conditions, leading to compartmentalization—a key step in the emergence of cellular life. Carbohydrates also played a fundamental role in prebiotic chemistry. They served not only as structural components of nucleic acids but also as essential molecules in primitive energy metabolism. Their synthesis and incorporation into early biochemical networks likely contributed to the development of metabolic pathways and the stabilization of informational polymers. Together, lipids and carbohydrates expanded the functional landscape of the prebiotic world, facilitating the transition from chemistry to biology.

The biophysical and biochemical properties of lipids render them indispensable in most contemporary OoL models. Understanding how lipids originated in the prebiotic environment, how the first micelles and vesicles formed, and how these structures evolved into selectively permeable membranes capable of supporting cellular life remains a central challenge in OoL research [86]. Current models generally propose that the formation of stable lipid bilayers began with the synthesis of monoacyl lipids (e.g., fatty acids and lysophospholipids), which were subsequently replaced by more complex diacyl lipids [87]. However, the non-enzymatic synthesis of diacyl phospholipids remains poorly understood. Notably, recent studies have demonstrated that natural glycerophospholipids can be synthesized non-enzymatically at 37 °C in aqueous conditions, offering a plausible prebiotic route for membrane formation [88]. A significant advancement in OoL research is represented by the GARD (Graded Autocatalysis Replication Domain) model. Supported by both computational simulations and experimental data, this model proposes that lipid-based assemblies could grow and divide while maintaining a self-replicating chemical composition—long before the emergence of nucleic acids or protein-based catalysis [89].

Carbohydrates, or sugars, are also indispensable for life, participating in essential metabolic pathways and serving as structural components of key biomolecules, including nucleic acids. Sugars such as glucose likely played a crucial role in energy production and the generation of metabolic intermediates necessary for the emergence of the first living cells. Pentoses like ribose were especially critical, as they formed the backbone of RNA, which could store information and catalyse reactions in early life forms. However, a major unresolved question is how pentoses could accumulate in sufficient quantities in the prebiotic environment, given their inherent chemical instability. One proposed solution involves a non-enzymatic pathway for five-carbon sugar synthesis in the prebiotic soup, based on chemical transformations analogous to the initial steps of the modern pentose phosphate pathway—a central route in contemporary metabolism [90]. These findings suggest that certain aspects of prebiotic metabolism may have closely resembled modern biochemical processes.

#### 6.1.2. The Supramolecular World

The supramolecular world refers to the organization of large, functionally significant molecular assemblies formed through non-covalent interactions among simpler molecular entities. This stage of prebiotic evolution likely succeeded the emergence of the biomolecular world, representing a higher level of structural complexity and molecular integration. Their emergence underscores the importance of self-organization, molecular cooperation, and structural integration in the progression from simple molecules to complex living systems.

Among the most significant supramolecular structures implicated in the OoL are membranes, ribosomes, and potentially viruses. These entities exemplify how molecular complexity can give rise to emergent properties essential for life, including compartmentalization, information processing, and replication. Membranes enabled the formation of protocells by providing selective barriers and spatial organization, thereby facilitating the concentration and regulation of biochemical reactions. Ribosomes, as ribonucleoprotein complexes, became central to the translation of genetic information into functional proteins. Viruses may have contributed to horizontal gene transfer and the evolution of early genetic systems, although their precise role in prebiotic evolution remains speculative.

An illustrative example of self-organization involving protein–DNA interactions in the formation of an infectious viral particle is the lambda (λ) bacteriophage, a double-stranded DNA virus widely utilized as a model system for investigating viral assembly mechanisms [91]. In vitro studies have demonstrated that its structural components—namely, capsid proteins and genomic DNA—are capable of spontaneous assembly under controlled physicochemical conditions. This process occurs independently of host cellular machinery, thereby providing a robust framework for elucidating the fundamental principles governing viral self-assembly.

Together, these supramolecular assemblies represent a pivotal transition from chemistry to biology, effectively bridging the gap between simple molecular interactions and the emergence of cellular life. Their formation marks a shift toward functional complexity and spatial organization, laying the groundwork for biological systems capable of replication, metabolism, and evolution.

##### Membranes

Cell membranes constitute the primary interface through which contemporary life maintains internal organization and mediates interactions with the external environment. In extant biological systems, membranes function as highly selective barriers, regulating the influx and efflux of substances. Beyond this role, they are integral to energy production via electrochemical gradients and to signal transduction through interactions with hormones and other stimuli. To fulfil these functions, membranes incorporate proteins within the lipid bilayer. In a prebiotic context, primitive membranes likely encapsulated genetic material along with other molecular components and metabolic precursors. This encapsulation would have conferred a significant selective advantage, ultimately leading to the emergence of the first protocells.

The appearance of closed yet selectively permeable compartments—membranes—was undoubtedly a pivotal milestone in OoL. Some theoretical models posit that the earliest evolvable life forms were already membrane-bound, whereas others suggest that initial compartmentalization occurred in membrane-free systems, such as rock pores, mineral surfaces, aerosols, coacervate droplets, or micelles [92]. Regardless of the initial mode of compartmentalization, the formation of membrane vesicles likely occurred early in the transition from prebiotic chemistry to primitive cellular life. These vesicles were probably formed by amphiphilic molecules, such as fatty acids, synthesized within the prebiotic milieu.

Laboratory experiments have demonstrated that amphiphilic compounds spontaneously self-assemble in aqueous environments into bilayer structures, which can close into spherical vesicles. These vesicles can encapsulate macromolecules—including nucleic acids and enzymes—thereby generating isolated microenvironments conducive to chemical evolution [93]. Such encapsulation was likely essential for the development of localized chemical reactions and the emergence of functional protocells.

According to the hypothesis proposed herein, the first fatty acids—likely short- or medium-chain—would have formed in the prebiotic soup. These amphiphilic molecules, either alone or in reaction with other compounds present in the same environment, could have led to the spontaneous assembly of vesicles. These vesicles, now part of the supramolecular domain, may have encapsulated all the necessary components for the formation of an incipient living system or protocell. Whether this prebiotic event occurred in freshwater environments or oceanic hydrothermal vents remains an open question [94]. It is reasonable to hypothesize that environmental fluctuations—including wet-dry cycles, pH and salinity variations, and temperature gradients—played a significant role in the evolution of these primitive protocells [95].

There is compelling evidence supporting the presence of short- and medium-chain fatty acids on the early Earth. Vesicles composed of such amphiphilic molecules have been shown to grow through lipid incorporation followed by division [96,97]. These replicating micelles or vesicles are capable of transmitting compositional information—encoded in their amphiphilic constituents or associated molecules—to subsequent generations. This mechanism enables a rudimentary form of natural selection and evolution, even in the absence of informational polymers such as RNA or DNA [97,98].

More recently, compartmentalization in aqueous solutions containing fatty acid mixtures (up to C19) has been demonstrated under reaction conditions compatible with Hadean Earth environments [99]. The resulting vesicle-like structures were capable of encapsulating fluorescent dyes, suggesting a plausible pathway for the formation of primitive membranes and protocells. This self-assembly process likely represents a critical step in the emergence of metabolism and life.

A recent study has demonstrated that the amino acid cysteine can spontaneously react with two short-chain (C8) thioesters to form diacyl lipids, resulting in the formation of membrane vesicles resembling protocells. This reaction occurs in aqueous conditions, and the resulting vesicles are compatible with functional ribozymes, suggesting that the coupling of multiple short-chain precursors may have provided membrane building blocks during early cellular evolution [100]. In our view, this experimental finding supports the existence of molecular worlds and their interactions. In this case, the interaction between cysteine and lipid molecules—initially belonging to distinct molecular worlds—results in the formation of membrane-like structures (belonging to the supramolecular world) potentially linked to the genesis of the first protocells.

##### Ribosomes

What components are required for a cell to synthesize proteins? In addition to the three major RNA types—mRNA, tRNA, rRNA—cells require the 20 standard amino acids, aminoacyl-tRNA synthetases, ATP, and various auxiliary molecular factors. Central to this process is the ribosome, a specialized organelle responsible for decoding genetic information and catalysing peptide bond formation. It is conceivable that the first ribosomes, or protoribosomes, formed spontaneously in the prebiotic broth, representing a key development in the biomolecular and supramolecular evolution of life. These protoribosomes may have arisen independently of translation, through structural affinities between specific RNAs and peptides, or they may have co-evolved with the protein synthesis process. They could represent the missing link between the RNA world and modern nucleic acid–protein-based life [101].

The ribosome, a ribonucleoprotein complex composed of RNA and proteins, serves as the molecular site where the genetic code is translated into functional proteins. As the universal machinery for protein synthesis in all living organisms, the ribosome also functions as a molecular fossil, offering critical insights into the OoL [81,102]. Structurally, it comprises a small subunit responsible for decoding mRNA and a large subunit that catalyses peptide bond formation at the peptidyl transferase centre.

In the prebiotic environment, it is plausible that ribozymes—catalytic RNA molecules originating from the RNA world—emerged first, capable of facilitating peptide bond formation and producing the earliest peptides. The vestige of such an ancestral ribozyme is now referred to as the protoribosome [103]. Crystallographic evidence supports the hypothesis that the earliest ribosomes lacked protein components, as the catalytic core of the modern ribosome—the peptidyl transferase centre—is composed entirely of RNA [104]. The association of this primitive ribozyme with peptides may have been favoured by the structural stabilization and compartmentalization provided by RNA, thereby facilitating the emergence of a prebiotic ribosome [105].

Experimental studies have demonstrated that under plausible prebiotic conditions, RNA molecules can independently catalyse the formation of short peptides [80]. Furthermore, when complementary RNA strands containing non-canonical base pairs are joined, the shared amino acids enhance the stability of the RNA duplex. These findings suggest a synergistic relationship between RNA and peptides in the prebiotic soup: RNA may have promoted peptide formation, while peptides may have stabilized and facilitated the elongation of RNA molecules. This mutual dependence mirrors the interdependence observed in modern biological systems, where RNA and proteins are required for each other’s synthesis—RNA polymerases synthesize RNA, and ribosomal RNA catalyses peptide bond formation. A key implication of this molecular synergy is that the central dogma of molecular biology—the directional flow of genetic information from nucleic acids to proteins—likely originated from a prolonged co-evolutionary process between RNA and peptides [106].

##### Viruses

Viruses occupy a distinctive position at the interface between living and non-living systems, rendering their biological classification a subject of ongoing scientific debate [107]. They are defined as acellular infectious agents composed of macromolecular complexes of proteins and nucleic acids. Within the framework of the recently proposed Lithbea domain—a taxonomic category designed to encompass entities that challenge traditional definitions of life, such as viruses and synthetic organisms—viruses are categorized as lifeless living systems [108].

Regardless of whether viruses are classified as living entities, their integral role in the biosphere is indisputable and they have profoundly influenced the evolutionary trajectory of life on Earth [109]. Viruses have contributed to evolution primarily through horizontal gene transfer, introducing genetic material across species boundaries. Viral infections also exert selective pressures, shaping immune system complexity and driving co-evolutionary dynamics, and endogenous viral elements, remnants of ancient infections, are embedded in many genomes and have been repurposed for essential biological functions. Viruses are not merely pathogens but key evolutionary agents that have helped shape the tree of life.

Viruses are increasingly considered relevant to discussions on the OoL. However, their direct involvement in the OoL remains speculative. Some hypotheses propose that viruses may have emerged prior to cellular life, acting as self-replicating genetic elements in a prebiotic world [110]. Notably, recent hypotheses propose that viruses may have been the progenitors of cellular life and the selfish catalysts of its evolutionary dynamics [111]. Furthermore, the virus-first theory presents a model in which viral lineages emerged before cells. Accordingly, viruses should be understood as a distinct class of ribonucleoprotein systems, some of which evolved directly from the ribonucleoprotein-world and contributed to the formation of cellular life via capsid evolution [112]. According with the AWH, viruses—or virus-like particles—emerged within the prebiotic milieu through interactions between RNA and proteins, forming primitive supramolecular assemblies with infectious potential. These protoviral entities may have persisted until the advent of cellular life or even contributed to the processes underlying the OoL. The most plausible scenario may be that certain viruses originated prior to the OoL, while others emerged later in conjunction with cellular evolution.

#### 6.1.3. The Metabolic World

Metabolism constitutes the intricate network of chemical reactions within living organisms, serving two fundamental purposes: the generation of energy required for essential biological functions and the biosynthesis of cellular components. For cellular survival and proliferation, a dynamic and efficient metabolic system is indispensable. It is therefore reasonable to propose that a primitive form of metabolism preceded the emergence of cellular life, operating within the prebiotic environment. This early metabolic activity would have ensured a continuous supply of molecular precursors necessary for the formation and maintenance of proto-biological systems [113]. This pre-cellular metabolic framework is herein referred to as the “metabolic world.”

Any robust model addressing the OoL must necessarily account for the emergence of metabolic networks that underpin the functionality of living systems. The universal presence of core biological features—such as the genetic code, mechanisms of genetic replication and expression, ATP synthesis via substrate-level phosphorylation and chemiosmosis, and central metabolic pathways strongly suggests that these systems originated at a very early stage in the evolutionary history of life.

It is plausible that the earliest metabolic pathways arose within the prebiotic milieu through the transformation of specific organic molecules into other compounds, which subsequently participated in further synthetic reactions. This cascade of molecular transformations gave rise to a protometabolism that laid the foundation for the metabolic world. Two studies are particularly relevant in this context: one examining the development of biochemical networks and biological complexity [114], and another investigating the evolution of the earliest metabolic cycles [115]. The emergence of a metabolic world, driven by the intrinsic chemical reactivity of atoms and molecules and shaped by physicochemical constraints, is supported by foundational studies on the prebiotic origin of ancient metabolic pathways predating enzymatic catalysis [54,59,116,117,118,119]. This metabolic world likely consisted of non-enzymatic, non-equilibrium reaction networks resembling modern central metabolic pathways, such as glycolysis, the pentose phosphate pathway, the Krebs cycle, the reductive acetyl-CoA pathway, and the glyoxylate cycle. In the absence of enzymes, these reactions were presumably catalysed by naturally occurring minerals containing transition metals such as Zn, Cr, and Fe. The metal-dependent enzymes found in extant organisms—such as cytochromes, nitrogenase, and ferredoxin—may represent evolutionary remnants of this prebiotic world, marking a transition from inorganic to organic catalysis [120]. Ribozymes present in the primordial environment may also have contributed to the formation of early metabolic pathways.

Several hypotheses have been proposed regarding the origin of the metabolic world. One prominent model suggests its emergence within hydrothermal systems, where metal ions—abundant in oceanic environments—could have catalysed key chemical reactions [121,122,123]. In this context, one of the most plausible scenarios for the OoL posits the existence of a prebiotic autotrophic metabolism within sulfide-rich hydrothermal vent environments. Empirical evidence indicates that hydrothermal vent chimneys can generate electron flow, and that such electron transport may have facilitated the abiotic synthesis of organic compounds in ancient deep-sea hydrothermal systems [124]. It has therefore been proposed that extensive metal production and metal-supported primordial metabolic processes likely arose as a natural consequence of the intense hydrothermal activity on the Hadean Earth [125]. Alternatively, another hypothesis favours a terrestrial geothermal setting, where wet–dry cycles could have driven thermodynamically unfavourable reactions [126]. Regardless of the specific environmental context—whether a deep-sea hydrothermal vent or a warm terrestrial pond—it is likely that the metabolic world emerged in concert with other prebiotic systems, and that components of these systems may have originated within this primitive metabolic framework.

A pivotal transition in the metabolic world was the shift from metal-based catalysis to enzyme-based catalysis. This transition was likely driven by several factors, including the toxicity of certain metals (e.g., iron), the superior substrate specificity of enzymes, and the capacity for enzymatic regulation. Enzyme-catalysed reactions are not only faster and more specific but also less harmful and amenable to regulation via allosteric mechanisms and covalent modifications [127].

An especially compelling hypothesis links the autotrophic biosynthesis of amino acids in the metabolic world with the emergence of the genetic code [123]. There is a biophysical basis for the establishment of the genetic code in protocells; for instance, correlations have been observed between the third base of codons and the size or hydrophobicity of the encoded amino acids [128]. In this context, the hypothesis proposed by N. Lane et al. is particularly noteworthy: they propose that patterns observed within the genetic code reflect direct biophysical interactions between amino acids and their cognate nucleotides, occurring within a hypothetical protometabolic environment such as autotrophically driven protocells. This interesting hypothesis offers a framework for understanding how and when the genetic code was established—a critical step in the emergence of the central dogma of molecular biology. In the context of the AWH proposed here, the OoL likely involved a convergence of the metabolic, RNA, and peptide/protein worlds, culminating in a primitive yet functional system capable of translating RNA sequences into proteins.

### 6.2. The Assembly Phase: Molecular Convergence and the First Living System

The second phase of the AWH posits that a variety of prebiotic molecular worlds, each with their own molecular compositions and dynamics, spontaneously evolved toward a common organizational structure. This convergence is not random but is guided by universal physical and chemical laws—such as thermodynamics, reaction kinetics, and molecular self-assembly principles. Through processes of self-organization, these systems collectively gave rise to the structured molecular assemblies that constituted the earliest living matter. In this context, molecular convergence refers to the tendency of chemically diverse prebiotic networks to evolve toward a shared organizational framework, ultimately enabling the emergence of a unified living system.

#### 6.2.1. The Concept of Molecular Convergence

The concept of molecular convergence, in the context of prebiotic chemistry and the OoL, refers to the process by which distinct molecular systems or worlds —each with their own structural and functional characteristics—gradually interact, integrate, and evolve toward greater complexity, ultimately contributing to the formation of a unified biological system that we call living system.

Initially, during the Priming Phase in the MEP, various molecular worlds may have emerged independently within a shared chemical environment. Each of these worlds would consist of molecules that are structurally and functionally related, interacting through catalytic, autocatalytic, or self-assembly mechanisms. For example, one molecular world might be dominated by lipid precursors, another by nucleotide derivatives, and another by amino acid-based polymers. These systems could operate semi-autonomously, undergoing internal transformations and generating increasing complexity within their respective domains.

Convergence occurred when these molecular worlds began to interact and influence one another. This interaction was facilitated by physical proximity, shared intermediates, and emergent compatibility between molecular components. Over time, such interactions would lead to the integration of previously separate processes—e.g., the coupling of informational molecules (like RNA) with catalytic molecules (like peptides), or the encapsulation of functional systems within lipid membranes. This integrative process was not instantaneous but gradual, driven by PDF.

The convergence of molecular worlds enables the formation of novel structures (e.g., protocells), emergent properties (e.g., compartmentalization, replication), and increasingly regulated networks of reactions. Ultimately, this convergence is what allows disparate molecular systems to coalesce into a coherent, self-sustaining entity—what we recognize as the first living system.

In summary, convergence is the transition from molecular diversity and compartmentalized functionality to integrated complexity and emergent biological organization. It is a central concept in understanding how life could arise from non-living chemical systems.

#### 6.2.2. The Genesis of the First Living System

During the transition from disordered living matter to the emergence of the first cellular entity, it is plausible that the constituents of the prebiotic milieu engaged in cooperative interactions, facilitating the rise in structural and functional order from an ostensibly chaotic environment. As self-organizing processes and self-sustaining reaction networks increased in complexity, molecular components involved in genetic expression, replication, energy production, and other essential functions became progressively integrated. This kind of molecular cooperation thus hypothetically played a pivotal role in the origin of the first cell, the formation of an ordered, membrane-bound structure—a thermodynamically open system capable of exchanging matter and energy with its surroundings. The progression from simple molecules to the first cellular forms involved multiple transitional stages [113,129], each contributing to an incremental increase in complexity, ultimately culminating in the OoL. In this context, we can see the transition from an ensemble of molecules to an ensemble of organisms—i.e., the OoL—as a special case of bona fide physical phase transitions associated with the emergence of a novel type of grand canonical ensemble and a corresponding new level of description [130].

From a physicochemical standpoint, the confinement of macromolecules within aqueous electrolytic environments promoted molecular interactions and fostered cellular self-organization [131]. Within this framework, RNA, DNA, ribosomes, and metabolic pathways co-evolved in a cooperative and organized manner within a membranous compartment, marking the transition from chemical evolution—characteristic of the prebiotic “cooking” phase—to biological evolution, which commenced with the OoL in the MEP and continues to the present. This initial cellular entity—here referred to as the Precursor of Biodiversity (PoB) cell—likely emerged following numerous unsuccessful attempts.

It is evident that the hypothesis I am proposing is not grounded in direct experimental evidence. Nonetheless, there are two conceptual arguments that may support this claim. First, it seems reasonable to assume that the emergence of the first living system required a membranous structure capable of enclosing the key components of biological activity. These would include the elements responsible for the storage and expression of genetic information, as well as a rudimentary metabolic system capable of generating energy and synthesizing molecules essential for sustaining the nascent life process. Second, as referenced through the text, several experimental studies demonstrate that, under plausible prebiotic conditions, all essential components of life—including membranes, RNA, peptides, ribosomes, and metabolic pathways resembling those found in contemporary organisms—could have emerged. If such components were enclosed within a semipermeable membrane and capable of self-organization, it is conceivable that a living system might have arisen. Admittedly, this remains speculative; I do not claim to know the answer, and it is fair to say that no one does. My perspective is informed both by specialized literature and by my own intuition as a biologist.

The convergence of these prebiotic worlds gave rise to a living system, with life arousing as an emergent property of the system. What was the biological nature of this primordial living system? If one adopts a functional definition of life [32,48], then the biological entity that arose from the prebiotic soup must have exhibited several defining characteristics. It would have constituted an organic system functioning as an entropy-producing, thermodynamically open system capable of exchanging matter and energy with its environment. Structurally and functionally self-organized, it would have performed metabolic processes essential for energy acquisition and biosynthesis, while also adapting to environmental fluctuations. Critically, it must have possessed a primitive genetic program encoding the minimal information required to regulate internal functions and enable replication and reproduction.

With the advent of this first living system, it began to manifest four essential attributes of the life process described above (Section 3): (1) the existence of biological information included within a mutable genetic program governing life and enabling evolutionary processes, (2) the establishment of the system–process duality; (3) the emergence of life as a consequence of the correct assembly of the living system’s components; (4) the generation of internal negative entropy necessary for self-organization, maintenance, and functioning of the primitive cell in a dynamic, non-equilibrium steady state. Collectively, these four attributes constitute the “vital factor”. Wherever this vital factor arises—whether on Earth, as in the OoL, or elsewhere in the universe—life will emerge, and its fundamental characteristics will be universally recognizable.

##### Autotrophs, Heterotrophs or Facultative Autotrophs?

Were the PoB cells autotrophic, heterotrophic, or did they exhibit metabolic flexibility indicative of facultative autotrophy? Current evidence from comparative genomics, metabolic reconstructions, and geochemical models suggests that the first cells were most likely chemoautotrophic. These ancestral cells are hypothesized to have derived energy from inorganic redox reactions—particularly involving hydrogen (H_2_) and carbon dioxide (CO_2_)—and to have fixed carbon via primitive autotrophic pathways, such as the reductive acetyl-CoA pathway [132,133,134]. This view aligns with the hydrothermal vent hypothesis, which posits that early life emerged in chemically rich, mineral-structured environments capable of sustaining autotrophic metabolism [123,135,136].

Current scientific consensus does not support the hypothesis that the earliest cells at the OoL were heterotrophic or facultatively autotrophic. Instead, prevailing models of primordial metabolism—particularly those based on alkaline hydrothermal vent environments—strongly favour an autotrophic last universal common ancestor (LUCA), which is thought to have harnessed energy through chemoautotrophic processes [137]. Heterotrophy is considered a later evolutionary development, arising independently in distinct lineages via convergent metabolic pathways [138]. Genomic reconstructions further support the notion that LUCA possessed a chemolithoautotrophic metabolism, utilizing molecular hydrogen and carbon dioxide, and likely thrived in serpentinizing hydrothermal systems [139]. Nonetheless, alternative scenarios remain plausible, and due to inherent limitations in reconstructing deep evolutionary history, it is unlikely that this question will ever be resolved with complete certainty.

As it was mentioned above, no current model strongly supports facultative autotrophy as the ancestral state. However, we would like to propose that PoB cells might be facultative autotrophs, i.e., an organism that could switch between autotrophic and heterotrophic modes of nutrition depending on environmental conditions. Given the environmental variability and the limited availability of organic substrates in early Earth conditions, it is plausible that PoB cells exhibited facultative autotrophy—a metabolic flexibility allowing them to switch between autotrophic and heterotrophic modes depending on resource availability. In fact, early Earth was highly dynamic, with fluctuating availability of energy sources and substrates. This hypothesis aligns with models suggesting that early metabolic networks were modular and adaptive, capable of exploiting both inorganic and organic compounds [132,140,141]. Facultative autotrophy may have represented a transitional stage in the evolution of metabolic complexity, preceding the specialization into strict autotrophic or heterotrophic lineages. Thus, while the precise nature of primitive metabolism remains uncertain, the facultative model offers a compelling framework for understanding the metabolic versatility that may have characterized the earliest living systems. It bridges the gap between purely physicochemical self-organization and the emergence of regulated, information-driven biochemistry.

## 7. The Onset of Biological Evolution

Once life originated on Earth and the first cell—or population of cells—emerged, evolution began its relentless course. The PoB cells initiated cellular division, leading to the expansion of the initial population. Concurrently, genetic mutations began to accumulate, while environmental pressures exerted selective forces that favoured the most advantageous cellular variants. From this point onward, natural selection assumed an increasingly pivotal role in the emergence of novel cellular entities, which would ultimately serve as precursors to both historical and contemporary biodiversity. Thus, the processes of reproduction and mutation within the original PoB population mark the inception of biological evolution. This entire evolutionary trajectory has been governed by physicochemical laws and the evolutionary forces. To these, we must now add the anthropogenic factor, as human activities are increasingly influencing the evolutionary pathways of numerous species and ecosystems.

Over an indeterminate span of time, this evolutionary process led to the emergence of the three living worlds: the Acellular World (AW), the Prokaryotic World (PW), and the Eukaryotic World (EW), each of which has continued to evolve to the present day. The interaction of these living worlds with the Environmental World (EnW) gave rise to ecosystems. The biosphere can thus be conceptualized as an endless spiral of life and evolution, continuously shaped by both natural and, more recently, anthropogenic forces [142,143,144].

### 7.1. A Framework for Biodiversity: The Four Interacting Worlds

An alternative framework for organizing biodiversity involves classifying all living entities into three distinct biological worlds: AW, PW, and EW. These worlds exist within and interact with the EnW, which comprises the abiotic substrate that sustains life [142,143]. AW primarily includes viruses, but also encompasses non-viral genetic elements such as viroids, plasmids, and transposons. While viruses are generally regarded as biological entities due to their capacity to infect and replicate within host cells, the aforementioned elements are typically excluded from the definition of living organisms. PW includes all prokaryotic life forms, classified into the domains Bacteria and Archaea. These organisms lack membrane-bound nuclei and exhibit extensive metabolic and ecological diversity, occupying a wide range of environmental niches and contributing significantly to biogeochemical cycles. EW encompasses all nucleated organisms and can be further subdivided based on nutritional strategies: autotrophs, which synthesize their own food via photosynthesis or chemosynthesis, and heterotrophs, which obtain energy by consuming other organisms or organic matter. This domain includes the vast diversity of unicellular and multicellular eukaryotes, from protists to plants, fungi, and animals. Finally, EnW refers to the abiotic components of the biosphere—air, water, soil, sunlight, temperature, climate, and mineral resources—that form the physical context in which biological processes and ecological interactions occur [142].

This four-world framework provides a comprehensive and integrative perspective on biodiversity, emphasizing the dynamic interplay between biological entities and their environmental substrate.

### 7.2. Evolutionary Forces

Evolutionary forces are the mechanisms that drive genetic change within populations over time and are fundamental to understanding the processes underlying biological evolution. Following the emergence of the first cell and the transition from a prebiotic environment to a primordial evolutionary context, these forces began to operate, ensuring the persistence of life and contributing to the development of biodiversity. Collectively, they provide a comprehensive framework for interpreting the complexity and directionality of evolutionary change. Five evolutionary forces have shaped the diversification of species:

1. Life Determinism, arising from the laws of physics and articulated through the Principle of Inexorability, posits that certain biological outcomes are inevitable given the constraints imposed by natural laws. This concept reflects a form of vital determinism observable across molecular to macroscopic scales.

2. Natural Selection, defined as the differential survival and reproduction of organisms best adapted to their environment, leads to the transmission of advantageous genetic traits to subsequent generations. It remains a central mechanism in shaping adaptive complexity.

3. Random Events, encompassing stochastic phenomena that are inherently unpredictable and uncontrollable, include mutations, genetic drift, and environmental disturbances. These events introduce variability and can significantly influence evolutionary trajectories, especially in small populations.

4. Interactions Between Worlds, referring to interspecies relationships and the dynamic formation and evolution of ecosystems. These interactions drive co-evolutionary processes and contribute to ecological complexity.

5. Anthropogenic Factor, a newly emergent evolutionary force that reflects the growing impact of human activities on the conservation, alteration, and generation of biodiversity. This includes habitat modification, climate change, pollution, and artificial selection, all of which are reshaping evolutionary pathways [144].

#### 7.2.1. Life Determinism: Deterministic Constraints in Evolution

Among the evolutionary forces shaping biodiversity, life determinism—conceptualized within the framework of the principle of inexorability [6]—can be understood as a direct consequence of the constraints imposed by physical, chemical, and biological laws. This principle asserts that many traits observed in living organisms and biological processes exist in their current form because no viable alternatives were feasible. In this context, certain characteristics are not merely the result of stochastic events but are instead shaped by deterministic necessities embedded in the structure of the universe. These constraints channel the course of biological evolution, ensuring that specific outcomes arise not solely through chance, but as inevitable consequences of natural law.

Life determinism also encompasses the concept of “predictable evolutionary pathways”, which suggests that biological complexity—including biodiversity and cellular organization—emerges through deterministic patterns shaped by environmental constraints, energy flows, and molecular interactions. This perspective implies that, under similar conditions, evolution may follow comparable trajectories, leading to the emergence of analogous biological structures or functions. However, it is important to clarify that this does not imply that evolution has a predetermined goal or direction. Evolution is inherently blind, driven by natural selection, genetic variation, and contingent historical events. It operates without foresight or intrinsic objectives. The notion of predictability refers to the underlying physical and chemical constraints that shape evolutionary possibilities, not to any teleological or purposeful progression.

Let us examine several instances of vital determinism as guided by the life determinism or the principle of inexorability.

Example 1: Wings. The emergence of wings is ultimately governed by the functional imperative of flight; no alternative structural solution fulfils this role with comparable efficacy. Natural selection acts upon random mutational variations, favouring individuals with increasingly efficient wing morphologies. Over evolutionary timescales, this process leads to the refinement of wing structures, enabling organisms to exploit aerial niches. This exemplifies physiological determinism, wherein the functional demands of flight necessitate the evolution of wings, and natural selection facilitates the proliferation of the most effective adaptations.

Example 2: Glycolysis. As previously discussed, glucose undergoes spontaneous conversion to pyruvate even in the absence of enzymatic catalysis, driven by a form of molecular determinism that favours this transformation. This reaction forms the basis of the universally conserved metabolic pathway known as glycolysis. Natural selection has optimized this process through the evolution of enzymes, allowing for its regulation and integration within broader metabolic networks. In this case, chemical determinism underlies the conversion of glucose to pyruvate, enabling ATP production and thereby sustaining cellular energy demands.

Example 3: Genetic code. The hypothesis of an informational determinism in the origin of the genetic code is a proposal I put forward here. Despite the theoretical possibility of alternative coding schemes, molecular evolution has consistently preserved the configuration we know. This persistence suggests that the genetic code may not be a product of arbitrary historical contingencies alone. While the precise mechanisms underlying its conservation remain unknown—and indeed, may be beyond current scientific understanding—it is plausible to consider that the genetic code reflects a form of biological informational determinism. Thus, the structure of the code may be constrained by deeper principles inherent to the nature of biological information processing, rather than being solely shaped by evolutionary chance. While the standard genetic code is nearly universal across all known life, there are documented examples of non-standard or variant genetic codes (mitochondrial genetic code, ciliate nuclear code, mycoplasma and other bacteria, archaea and some protists). These examples show that while the genetic code is highly conserved, it is not absolutely fixed. However, the rarity and limited scope of these variations reinforce the idea that the standard code is under strong evolutionary constraints—possibly supporting the hypothesis of biological informational determinism.

Example 4: Eye structure. The convergent evolution of camera-like eyes in vertebrates and cephalopods illustrates how optical determinism governs the design of visual systems. Despite their independent evolutionary origins, both lineages arrived at similar structural solutions—lens, retina, and photoreceptor arrangement—because these components represent the most effective configuration for image formation under the laws of optics.

Example 5: Bipedal locomotion in humans. The evolution of bipedalism in hominins can be interpreted through mechanical determinism. Given the anatomical constraints and environmental pressures, upright walking emerged as the most energy-efficient mode of terrestrial locomotion. The restructuring of the pelvis, spine, and lower limbs reflects adaptations that were not arbitrary but dictated by biomechanical principles.

These examples illustrate the interplay between deterministic constraints and evolutionary mechanisms, emphasizing how natural selection operates within the boundaries defined by physical and chemical laws.

#### 7.2.2. Natural Selection

Natural selection is a fundamental mechanism of evolutionary change, first articulated by Charles Darwin in On the Origin of Species [145]. It refers to the differential survival and reproductive success of phenotypes that exhibit superior adaptation to their environmental conditions or specific ecological contexts within a biological population [146]. It operates on the principle that individuals within a population exhibit variation in traits, some of which confer differential survival or reproductive advantages in a given environment. These advantageous traits are more likely to be passed on to subsequent generations, leading to their increased prevalence over time.

The role of natural selection in shaping species evolution represents a foundational and extensively corroborated principle within the biological sciences. This evolutionary force acts on phenotypic variation arising from genetic mutations, recombination, and other sources, favouring traits that enhance fitness. Importantly, while the process is driven by random genetic variation, the selection itself is non-random, guided by environmental pressures and functional demands.

Given its well-established position in evolutionary theory, a detailed analysis of this mechanism falls outside the scope of the present study.

#### 7.2.3. Random Events

In this context, random events refer to inherently stochastic phenomena—those that are unpredictable and beyond human control. Among biological processes, mutations constitute spontaneous alterations in genetic material that introduce novel genetic variants, thereby supplying the raw material for evolutionary change [147,148]. Additionally, genetic drift and gene flow—resulting from the movement of individuals between populations—are key stochastic mechanisms that shape patterns of genetic diversity [149]. Genetic drift refers to random changes in allele frequencies within a population, particularly pronounced in small populations. These changes are not driven by natural selection but occur due to chance events—such as which individuals happen to reproduce. Gene flow, also known as migration, is the transfer of genetic material between populations through the movement of individuals or their gametes.

Beyond biological factors, random physical events also exert a significant influence on evolutionary trajectories. Geological phenomena such as volcanic eruptions, earthquakes, and plate tectonics, along with climatic fluctuations involving changes in temperature, precipitation, and sea levels, can dramatically reshape landscapes and create new ecological niches [150,151]. These events may lead to population isolation, trigger migrations, or cause extinctions, thereby influencing both the direction and pace of evolutionary change. Ultimately, the tempo of evolution is closely linked to the dynamic physical transformations of the Earth.

#### 7.2.4. Interactions Between Worlds

Within ecosystems, a complex web of interdependence exists among organisms from the acellular, prokaryotic, and eukaryotic worlds. These systems encompass numerous interactions both within and across biological worlds, as well as between these domains and their abiotic environments [142,143]. Viruses exemplify such interworld interactions, engaging with acellular, prokaryotic, and eukaryotic entities. Although these interactions may initially appear strictly parasitic—given that viruses exploit host cellular machinery for replication, often culminating in host cell death—they have played a pivotal role in the evolution of life on Earth. Viral activity has contributed to horizontal gene transfer, the emergence of diverse diseases, and the regulation of microbial populations.

Another form of interworld interaction is mutualism, as observed in cooperative relationships between bacteria and eukaryotic organisms. Prominent examples include the symbiosis between nitrogen-fixing bacteria and leguminous plants, as well as the microbial communities residing in the human gastrointestinal tract. A third type of ecological interaction is the predator-prey dynamic, which is essential for maintaining population equilibrium among various organisms. Furthermore, environmental disturbances—such as droughts, wildfires, or other abiotic stressors—can significantly modulate these ecological relationships.

These interactions across biological worlds are considered emergent phenomena, arising from the formation and evolutionary development of ecosystems. Importantly, beyond naturally occurring interactions shaped by evolutionary processes, anthropogenic activities have introduced novel forms of interdependence, further altering ecological dynamics.

#### 7.2.5. The Anthropogenic Factor

The anthropogenic factor encompasses the influence of human activities and population growth on the structure and dynamics of ecosystems [144]. An ecosystem is a complex, interactive, and open system of life embedded within a specific environment, whose evolution is governed by natural laws. However, this equilibrium has been profoundly disrupted by the emergence of humans as the planet’s dominant species. Human actions have significantly reshaped the architecture and functionality of ecological networks through multiple pathways, including the introduction of invasive species and synthetic organisms, environmental pollution, landscape transformation, illegal hunting and fishing, and the overexploitation of natural resources. Moreover, the continuous expansion of the human population contributes to habitat degradation and pose substantial challenges to the conservation and protection of ecosystems and biodiversity.

## 8. The Creation of Biodiversity

The history of life on Earth represents a continuous evolutionary trajectory, tracing the transition from non-living matter to the emergence of biological diversity. From the moment life first appeared, evolutionary mechanisms began to operate, giving rise to novel life forms that, through their integration into the environment, contributed to the formation of the earliest ecosystems. Once established, these ecosystems themselves evolved in response to changing environmental conditions.

This evolutionary journey—characterized by both progress and setbacks and shaped by challenges both surmountable and insurmountable—has unfolded over millions of years. Throughout this process, numerous species have disappeared, while others have emerged and adapted to new biological and environmental contexts. The theory of evolution provides a foundational framework for understanding these transformations, governed by the laws of nature [152], the stochastic influence of chance [153], and the intrinsic principles of life [6].

### 8.1. Beyond LUCA: The PoB Cells

LUCA is widely recognized by the scientific community as the hypothetical organism from which all extant cellular life forms are descended [154]. It represents the ancestral cell that gave rise to both Bacteria and Archaea, from which eukaryotic organisms subsequently evolved. Recent studies estimate that LUCA existed approximately 4.2 billion years ago and possessed a genome of at least 2.5 megabases, comparable in size to that of modern prokaryotes [155,156].

The traditional three-domain model of the tree of life—comprising Archaea, Bacteria, and Eukarya—positions LUCA as the common ancestor of all known life forms. More recent models, such as the two-domain hypothesis, propose that eukaryotes emerged from within the archaeal domain, yet still place LUCA at the root of the evolutionary tree [157]. Archaea exhibit features shared with Bacteria, including prokaryotic ribosomes, circular chromosomes, and the absence of membrane-bound organelles. However, they also possess traits in common with Eukaryotes, such as DNA associated with histones, multiple types of RNA polymerases, and the use of methionine as the initiating amino acid in protein synthesis [158]. Current evidence suggests that eukaryotes evolved from a lineage within the archaeal superphylum Asgard [159], and the existence of a Last Eukaryotic Common Ancestor (LECA) has been proposed to explain the origin of eukaryotic organisms within the archaeal domain [160].

Once life originated on Earth, over an indeterminate span of millions of years, both cellular and acellular living worlds came into existence. A central question in this context is: how did these distinct living worlds originate? While the existence of LUCA is supported by substantial scientific evidence, it remains a theoretical construct. Its primary value lies in providing a conceptual framework for reconstructing the evolutionary history of life and understanding the emergence of biological diversity. Nonetheless, alternative hypotheses regarding the origins of prokaryotic and eukaryotic life remain plausible and merit consideration.

#### PoB Cells

In the hypothesis proposed herein, life emerged within a prebiotic environment as a population of primitive and unstable cellular entities, referred to as PoB cells. It is important to underscore that these early forms are conceptualized not as a singular common ancestor—such as LUCA—but rather as a heterogeneous pool of primordial cellular structures. From this diverse population, at least two distinct evolutionary lineages are hypothesized to have arisen, ultimately giving rise to the domains now recognized as Prokaryota and Eukaryota.

PoB cells designates a theoretical class of primordial biological entities that may have represented an early transitional stage in the emergence of cellular life. These cells are hypothesized to have contained genetic material—likely RNA and primitive forms of DNA—encoding essential proteins required for fundamental biological functions. These include energy metabolism, possibly via rudimentary glycolytic or fermentative pathways; biosynthesis of key biomolecules such as monosaccharides, amino acids, nucleotides, and lipids; and membrane formation and maintenance, enabling compartmentalization and selective exchange with the environment. Furthermore, PoB cells are proposed to be facultative autotrophs, exhibiting a degree of biochemical autonomy that allowed them to sustain life processes independently within their environmental context.

PoB cells would have crossed the threshold into biological life by acquiring genetic continuity through replication, catalytic activity via encoded proteins and/or ribozymes, and selective permeability and homeostasis, thereby enabling interaction with and adaptation to their surroundings. Collectively, these features suggest that PoB cells may have constituted self-replicating systems with a minimal yet functional genome, analogous in some respects to the theoretical attributes ascribed to LUCA.

The emergence of PoB cells marks a pivotal transition from prebiotic chemistry to biological evolution. Their existence implies a stage at which natural selection could act upon molecular systems, favouring those with enhanced stability, replication fidelity, and metabolic efficiency. Over time, these PoB cells may have diversified, giving rise to distinct lineages that ultimately led to the domains of Bacteria, Archaea, and Eukarya. Understanding PoB cells is relevant not only to evolutionary biology but also to synthetic biology, where efforts are underway to reconstruct minimal cells or engineer novel life forms. Insights into PoB-like systems could inform the design of artificial cells capable of autonomous function, thereby illuminating the origin of life and the boundary between chemistry and biology.

### 8.2. The Roots of Biodiversity

**Figure 3:**
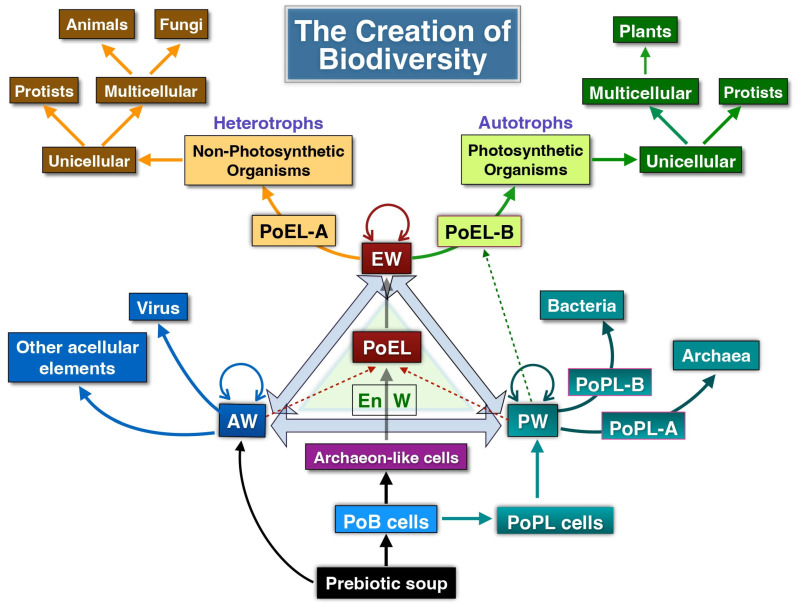
The creation of biodiversity. In the prebiotic soup, the molecular prebiotic worlds gradually disappeared as the earliest living systems began to form. From that primordial era, only viruses likely persisted, co-evolving alongside the emergence of prokaryotic and eukaryotic biodiversity. The first living systems on Earth are referred to as PoB cells. From this original population, two major subpopulations emerged: PoPL cells and archaeon-like cells, which, respectively, gave rise to the prokaryotic and eukaryotic domains. Within the PoPL subpopulation, two distinct cell types can be identified: PoPL-B cells, which were precursors to bacteria, and PoPL-A cells, which led to the archaeal lineage. Archaeon-like cells—closely resembling or even identical to PoPL-A cells—eventually evolved into PoEL cells. The structural and functional similarities between archaeon-like cells and PoPL-A cells would explain why archaea exhibit features common to both bacteria and eukaryotes. Two pivotal events marked the transition from primitive prokaryotic cells to the first eukaryotic cells: the acquisition of mitochondria, widely explained by the endosymbiotic theory, and the emergence of the nuclear membrane, whose origin remains a subject of ongoing scientific debate. The appearance of eukaryotic life likely involved contributions from both the PW and possibly the AW, particularly through early viral interactions. A lineage of PoEL cells gave rise to PoEL-A cells, which evolved into the first unicellular heterotrophic organisms. These organisms eventually diversified into the animal kingdom and the fungal domain—initially as unicellular forms and later, through cooperative processes, as multicellular organisms. Meanwhile, PoEL-B cells gave rise to the first photosynthetic organisms. A key step in this transition was the acquisition of a photosynthetic organelle, likely through another endosymbiotic event analogous to mitochondrial acquisition. This marked the origin of autotrophic life and the beginning of the plant kingdom, starting with unicellular algae and evolving into lichens and vascular plants. The entire evolutionary process was governed by three fundamental forces: the laws of nature (physics and chemistry), the commandments of life [6], and evolutionary forces (see main text). In the 21st century and beyond, an additional factor must be considered: the anthropogenic factor, referring to the impact of human activity and overpopulation on biodiversity and ecosystems. In the associated figure, the triangle at the center represents the ecosystem, with its vertices corresponding to the three biological worlds. The green triangle at the center symbolizes the physical space (EnW) where life unfolds. The central placement of the ecosystem emphasizes that all processes leading to life and biodiversity occurred within ecological contexts. Evolution and ecosystems are inseparable concepts in understanding the origin and development of life on Earth. The double curved arrows in the figure represent interactions among species within the same biological world. Thick double arrows indicate interactions between different worlds. Dashed red arrows suggest possible contributions from the prokaryotic and acellular worlds to the genesis of the first eukaryotic cell. The dashed green arrow represents the endosymbiotic event that led to the emergence of the first photosynthetic organisms.

There exists a broad scientific consensus concerning the general structure of the tree of life, and genomic data have substantially advanced our understanding of interspecific evolutionary relationships [161]. Nevertheless, despite the increasing robustness of phylogenetic and metagenomic analyses, their conclusions should not be considered definitive. The origin and early evolution of life may have proceeded in ways that current phylogenetic models do not fully encompass. Phylogenetic trees derived from DNA or RNA sequences reflect shared ancestry but do not necessarily elucidate the nature of the earliest living entities that emerged following the OoL. In this regard, other cellular and molecular characteristics—such as the presence of a nucleus, the universality of the genetic code and central metabolic pathways, membrane composition, and energy transduction mechanisms—are equally essential for understanding both the OoL and the emergence of biodiversity. Figure 3 shows a visual representation of the evolutionary tree of life as proposed in this work.

With respect to acellular life, as previously discussed, it is plausible that the earliest viruses originated within the prebiotic environment, forming part of a primordial world composed of proteins and RNA. This suggests the existence of an initial AW that may have preceded the emergence of cellular life. Subsequently, novel RNA and DNA viruses could have evolved in association with the diversification of cellular organisms. The implication is that viral entities may have played a foundational role in early molecular evolution, potentially contributing to the development of genetic exchange mechanisms and influencing the trajectory of biological complexity.

With regard to cellular life, the hypothesis proposed herein suggests that two distinct lineages emerged from a primordial pool of PoB cells: one giving rise to the PW and the other to the EW (Figure 3). Contrary to the prevailing view that eukaryotes evolved from a subset of archaea, it is posited that the precursor cells of prokaryotic life (PoPL) and those of eukaryotic life (PoEL) originated independently from PoB cells. The PoPL lineage subsequently diverged into two distinct cellular branches: PoPL-B, which led to the domain Bacteria, and PoPL-A, which led to the domain Archaea. The existence of these two types of PoPL cells provides a conceptual framework for understanding both the shared features and the profound differences observed between archaea and bacteria.

The rationale for distinguishing the origins of prokaryotic and eukaryotic organisms is based primarily on the absence of a nucleus in prokaryotes—a structural feature considered here to be more defining than phylogenetic relationships inferred from DNA sequence analyses. While this hypothesis cannot be empirically validated, it is important to note that the archaeal-origin hypothesis for eukaryotes likewise lacks definitive empirical support. In this domain of inquiry, we must necessarily rely on conjectures grounded in current empirical knowledge and theoretical plausibility.

While PoPL cells evolved into the prokaryotic domains, other PoB cells—sharing certain features with PoPL-A and thus referred to as archaeon-like cells in Figure 3—are hypothesized to have evolved into PoEL cells, the precursors of eukaryotic life. Two major evolutionary events were pivotal in this transition: the acquisition of mitochondria and the formation of a nucleus to compartmentalize genetic material. The endosymbiotic theory accounts for the origin of mitochondria and chloroplasts, proposing that these organelles evolved from free-living prokaryotes that entered into symbiotic relationships with ancestral eukaryotic cells [162]. Phylogenetic analyses, along with logical inference from observable cellular structures, suggest that the evolution of mitochondria and chloroplasts occurred at distinct temporal points [163].

In contrast, the origin of the nucleus remains a subject of considerable debate. To explain this enigmatic transition, two principal hypotheses have been proposed: (i) the nucleus originated via a symbiotic event, or (ii) it emerged through internal remodeling of an ancestral cell [164,165,166]. The archaeal origin of many components of the eukaryotic information-processing machinery, along with studies of archaeal lineages such as *Lokiarchaeia* and the TACK superphylum (which includes *Thaumarchaeota*, *Aigarchaeota*, *Crenarchaeota*, and *Korarchaeota*), supports a model in which the nucleus evolved from an archaeal ancestor [167]. An alternative hypothesis posits that the nucleus originated from the fusion of two ancient archaeon-like cells [168], potentially combining distinct genetic and metabolic capabilities.

Although the fusion model offers a framework for understanding the complex architecture and dual genetic heritage observed in modern eukaryotic cells, it is worth considering a fundamentally different hypothesis: that the nucleus descends from a DNA virus that infected the archaeal ancestor of eukaryotes [169,170]. Notably, a recent study demonstrated the formation of a nucleus-like compartment during viral infection of a bacterium, wherein proteins involved in DNA replication and transcription were localized within the compartment, while those involved in translation and nucleotide synthesis remained external [171]. This observation provides compelling support for the viral origin hypothesis, suggesting that viruses may have pioneered the spatial separation of genetic processes.

In our view, such findings significantly strengthen the plausibility of this model, implying that viral mechanisms could have played a foundational role in the compartmentalization of genetic functions during early eukaryogenesis. This perspective not only challenges traditional views of cellular evolution but also highlights the potential influence of acellular life forms in shaping the architecture of complex cells.

The evolutionary transition from archaea to eukaryotes remains poorly understood, primarily due to the absence of cultured representatives and the limited physiological data available. A study published in recent years may provide evidence for the existence of PoEL cells. In that work, the authors reported the decade-long isolation of an Asgard archaeon affiliated with Lokiarchaeota from deep marine sediments [172]. This microorganism exhibits a distinctive metabolism and morphology, leading the authors to propose the entangle–engulf–endogenize model as a conceptual framework for explaining the emergence of eukaryotes.

PoEL cells ultimately gave rise to the eukaryotic domain, which was initially composed of unicellular organisms. Over time, some of these organisms formed cooperative associations, leading to the emergence of multicellularity and the development of cellular specialization, tissues, and organs. A pivotal event in the evolution of the plant lineage was the acquisition of chloroplasts, most likely through a process analogous to mitochondrial endosymbiosis. Over millions of years, PoEL cells diversified into two major functional groups: photosynthetic autotrophs—including plants and unicellular algae—and heterotrophs, encompassing protists, animals, and fungi.

Multicellularity constitutes one of the major evolutionary transitions in the history of life. Its defining feature is cooperative integration, which not only facilitated the colonization of diverse biotopes by a wide array of species but also drove an increase in organismal complexity—manifested in the organization of distinct cell types into tissues and organs during morphogenesis. Although the actual tree of life [161,173] is considerably more complex than the schematic representation in Figure 3, a comprehensive account of all prokaryotic and eukaryotic branches lies beyond the scope of this paper.

### 8.3. Ecosystems and Cooperativity

Throughout the evolutionary process that led to the emergence of biodiversity, beyond the mechanisms that generate genetic variability and provide the substrate for natural selection, three additional elements have played a fundamental role: the interactions within the ecosystem, cooperative phenomena, and horizontal gene transfer (HGT).

In the tree of life depicted in Figure 3, past and present biodiversity is organized around the core of the figure, which is occupied by a triangle symbolizing the ecosystem. This representation emphasizes the dynamic interactions among the biological worlds (AW, PW, EW) and their shared environment. The arrows not only illustrate the interactions between organisms and their abiotic surroundings but also reflect the multitude of cooperative relationships—both positive and negative—that are essential for maintaining ecological balance [6,174,175].

Cooperation is ubiquitous in nature, manifesting at all levels of biological organization, from molecular interactions to complex symbiotic and social behaviours. Cooperative phenomena were instrumental in two of the most transformative events in the history of life: the dawn of eukaryogenesis [176] and the emergence of multicellularity. Since then, cooperative phenomena at all levels of complexity, from molecules to ecosystems, have been essential to the evolution of species. For instance, at the molecular level, cooperation is evident in metabolic networks, gene expression systems, and signal transduction pathways. It is also evident in human societies and in social insects. A notable example of microbial cooperation is the formation of biofilms—structured microbial communities that adhere to surfaces and exhibit a primitive form of multicellularity as a survival strategy [177,178].

Symbiosis, broadly defined as any close and long-term biological interaction between members of different species, is now recognized as one of the primary forces shaping life on Earth [179]. Numerous examples of symbiotic cooperation can be observed today, involving species from the same or different biological domains. These include coral reefs, mutualisms between ants and fungi, lichens, bioluminescent organs, gut microbiota, sea anemones and hermit crabs, African oxpeckers, nitrogen-fixing bacteria, and holobionts, among others [180,181,182]. The increasing complexity of multicellular organisms necessitated the evolution of coordination mechanisms to ensure cooperation among cells, tissues, and organs. Life cycles and life histories can readily emerge at the origin of multicellularity, shaped by ancestral constraints and ecological conditions [183].

HGT defined as the non-reproductive transmission of genetic material between organisms not linked by a parent-offspring relationship [184,185], represents another crucial mechanism in the evolution of life. HGT has significantly shaped the web of life by enabling gene exchange among prokaryotes, between prokaryotes and eukaryotes, and even among multicellular eukaryotes. Notably, viruses have also played a pivotal role in mediating HGT to their hosts [186].

## 9. Perspectives on the Future of Life Research

What does the future hold for our understanding of life? Will we one day be able to synthesize a living cell from inert matter in a test tube? Is there life beyond Earth? How will the growing influence of our species on the biosphere shape the future of life on our planet? What novel organisms might be created in our laboratories? These are among the many questions that future scientific inquiry may help to address.

Looking ahead, the principal theoretical and experimental challenges in the study of life and living systems can be broadly categorized into three issues:

1. In Vitro Life Creation: Achieving the synthesis of life from non-living matter would represent a major milestone in both conceptual understanding and methodological innovation.
2. Advancements in Synthetic Biology: The engineering of novel biological systems holds the potential for transformative applications, while simultaneously posing significant ecological and bioethical risks.
3. The Search for Extraterrestrial Life: The discovery of life beyond Earth would raise fundamental questions about the universality of biological principles and the diversity of life forms in the cosmos.

The synthesis of life from abiotic components remains one of the most profound and ambitious goals in contemporary science. This endeavour seeks not merely to replicate biological functions, but to elucidate and reconstruct the minimal conditions required for life to emerge. The concept of creating life in a test tube involves assembling all molecular constituents necessary for an entity to be considered a living system. The implications of such an achievement would be far-reaching: it would offer unprecedented insights into the OoL, define the minimal requirements for cellular existence, enable the development of novel biological systems and facilitate the design of organisms capable of surviving in extraterrestrial environments. This pursuit also raises critical ethical and philosophical questions regarding the nature of life and humanity’s role as creators of living systems.

Synthetic biology integrates principles from biology, engineering, computer science, and chemistry to design and construct new biological systems or reprogram existing ones. A central objective of this field is the rational design of biological components that can be assembled into functional modules and incorporated into living cells to perform specific tasks. Genetically modified organisms (GMOs) exemplify genomic manipulation for diverse purposes, including agricultural enhancement and the production of therapeutically relevant compounds. The advent of genetic toolkits such as CRISPR-Cas systems has revolutionized genome editing, enabling precise and efficient modifications across a wide range of organisms. Other key areas of investigation include the construction of minimal cells, the design of biosynthetic pathways, and the development of organisms with expanded genetic codes [187,188]. The integration of artificial intelligence (AI) and machine learning into synthetic biology is further accelerating progress, enhancing our capacity to model, predict, and optimize biological designs. A striking example of this convergence is the creation of xenobots—living, self-powered robots constructed from frog stem cells, designed computationally and assembled in vitro—representing a novel form of synthetic life [189].

These remarkable advances also necessitate careful consideration of ethical, safety, and regulatory frameworks. The potential for unintended consequences, ecological disruption, or misuse of synthetic organisms underscores the need for robust risk assessment and governance. Once released, synthetic organisms may evolve unpredictably, potentially acquiring traits that render them harmful or invasive. Additionally, the possibility of malicious use—such as the engineering of novel pathogens—cannot be dismissed. Unequal access to synthetic biology technologies may further exacerbate global disparities in health, agriculture, and industry.

The question of whether life exists beyond Earth remains one of the most profound and enduring mysteries in science. Although direct evidence of extraterrestrial life has yet to be discovered, the identification of extremophiles on Earth, the detection of potentially habitable exoplanets, and the presence of biomolecules in meteorites and interplanetary dust have strengthened the hypothesis that life may be widespread in the universe [190,191]. Understanding the nature of such life forms—should they exist—is essential not only for astrobiology but also for anticipating the biological, technological, and ethical implications of potential contact.

What might extraterrestrial organisms be like? My position is that such life forms would likely resemble those known on Earth. This assertion is based on three fundamental premises: (i) the laws of physics and chemistry are presumed to be universal; (ii) the periodic table of elements is consistent throughout the cosmos; and (iii) organic molecules and biomolecules have been detected in cosmic material that reaches our planet. Could alternative biochemical systems—those not based on carbon chemistry—give rise to different forms of life? The honest answer is that we do not know. However, it is certain that all known life is based on carbon chemistry. Beyond this, any speculation remains purely hypothetical. Furthermore, by applying the principle of inexorability, one might infer that if flying organisms exist on another planet, they would require wings; if they needed to perceive their environment visually, they would possess eyes. This principle may even extend to the molecular level—for example, the synthesis of ATP would likely occur via substrate-level phosphorylation or chemiosmotic mechanisms, as these are the most efficient energy conversion processes known. Although the existence of extraterrestrial life remains hypothetical, its eventual discovery—whether microbial or intelligent—could profoundly expand the boundaries of human knowledge and reveal novel adaptations to environments and climates unlike those on Earth.

## 10. Final Remarks

The challenge of defining life and uncovering its origins is not solely a scientific endeavour, but also a conceptual and philosophical one. It requires a synthesis of empirical evidence, theoretical rigor, and reflective analysis. As research progresses, it becomes increasingly clear that the questions of what life is and how it began are deeply interdependent. A clearer understanding of one inevitably refines our perspective on the other. In this pursuit, interdisciplinary collaboration and open-ended inquiry are essential to deepening our understanding of life’s fundamental nature.

Although this work may initially appear to encompass disparate elements, it is unified by a central theme: life. From its origin to the diversification of biodiversity across geological timescales, this study aims to present a coherent narrative. Within this framework, the anthropogenic factor emerges as increasingly significant, as human activity now plays a dominant role in shaping the future of biodiversity. Paradoxically, it is also through human innovation and technological advancement that we may gain deeper insights into life’s origins on Earth and into the possibility of similar processes occurring elsewhere in the universe.

While this work draws on experimental findings from the scientific literature, it remains inherently speculative—like many contributions in this field—since it does not claim to reconstruct past events with certainty. Were such certainty attainable, the nature of this inquiry would be fundamentally different. It is worth signalling that many foundational principles in physics and biology began as speculative ideas, without diminishing their scientific value or transformative potential.

Not all readers will agree with the hypothesis presented here. I do not claim to possess a definitive answer to the OoL—nor does anyone else—and it is likely that we may never fully know what transpired in the distant past. The aim of this work is not to assert certainty, but to offer a perspective on how life and biodiversity may have emerged on this planet. Ultimately, time and scientific inquiry will determine the merit of this view.

## Data Availability

Not applicable.

## Abbreviations

The following abbreviations are used in this manuscript:

AI	Artificial Intelligence
AW	Acellular World
AWH	Assembled World Hypothesis
EW	Eukaryotic World
EnW	Environmental World
HGT	Horizontal Gene Transfer
LUCA	Last Universal Common Ancestor
MEP	Mysterious Earthly Place
OoL	Origin of Life
PCR	Polymerase Chain Reaction
PDF	Physicochemical Driving Forces
PoB	Precursor of Biodiversity
PoPL	Precursor of Prokaryotic Life
PoEL	Precursor of Eukaryotic Life
PW	Prokaryotic World
